# The cyclin-dependent kinase inhibitor p27 facilitates chemosensitivity by promoting ferroptosis in epithelial ovarian cancer

**DOI:** 10.1016/j.jbc.2025.111011

**Published:** 2025-12-05

**Authors:** Mengna Zhu, Lin Huang, Si Sun, Mengqing Chen, Jing Cai, Yuan Zhang, Lufang Wang, Liqiong Cai, Minggang Peng

**Affiliations:** 1 Department of Obstetrics and Gynecology, Union Hospital, Tongji Medical College, Huazhong University of Science and Technology, Wuhan, China; 2 Chinese Institutes for Medical Research, Capital Medical University, Beijing, China

**Keywords:** CDKN1B, chemoresistance, cytochrome b-245 heavy chain, iron-dependent cell death, ovarian carcinoma

## Abstract

Platinum-based chemotherapy remains the cornerstone of the treatment of epithelial ovarian cancer (EOC); however, platinum resistance is a major cause of tumor recurrence and mortality in EOC patients. This study reports the mechanism by which EOC cells develop resistance to cisplatin by inhibiting the ferroptosis. Immunohistochemical analysis of human EOC tissues revealed that low p27 expression significantly correlated with poor response to chemotherapy and unfavorable patient prognosis. Functionally, CRISPR/Cas9-mediated p27 KO increased cisplatin resistance in EOC cells in both *in vitro* and *in vivo* models. RNA sequencing and functional assays demonstrated that p27 enhanced cisplatin sensitivity by facilitating drug-induced ferroptosis in EOC cells. Mechanistically, luciferase reporter assays demonstrated that p27 enhanced the transcriptional activity of cytochrome b-245 heavy chain, a key positive regulator of ferroptosis. Moreover, we found that the small molecule inhibitor SKPin C1 significantly enhanced cisplatin’s antitumor effect by preventing p27 degradation, both *in vitro* and *in vivo*. In conclusion, our findings emphasize the critical role of p27 in triggering ferroptosis, thereby sensitizing EOC cells to cisplatin therapy. These results suggest that therapeutic strategies aimed at enhancing p27 levels may represent a promising approach to overcoming chemoresistance in EOC.

Epithelial ovarian cancer (EOC) is the second most lethal gynecologic malignancy and ranks as the eighth leading cause of cancer-related deaths among women globally (1). The standard treatment for EOC patients consists of primary debulking surgery followed by platinum-based chemotherapy (2). Despite an initial response rate of 75 to 80% to first-line platinum-based chemotherapy, approximately 70% of patients experience relapse and ultimately develop resistance to platinum-based treatment, resulting in therapeutic failure (3, 4). Therefore, elucidating the molecular mechanisms underlying chemotherapy resistance is crucial for the development of therapeutic strategies that can enhance the efficacy of treatment in EOC patients.

Cisplatin is one of the most widely used chemotherapeutic agents in the treatment of various solid tumors, including ovarian, breast, testicular, lung, and others (5). Traditionally, the cytotoxic effects of cisplatin have been attributed to its ability to form DNA adducts, leading to DNA damage and triggering apoptosis (6). However, recent studies have identified that ferroptosis, a regulated form of cell death, is also a critical mechanism of cisplatin-induced cytotoxicity. Ferroptosis is driven by the accumulation of iron-dependent lipid peroxidation, which disrupts the plasma membrane integrity and generates high levels of reactive oxygen species (ROS) (7). Cisplatin induces ferroptosis by disturbing the intracellular redox balance, particularly through targeting the GSH-glutathione peroxidase system (8). Despite this, many cancer cells develop intricate mechanisms to evade cell death, contributing to drug resistance and tumor progression (9). Although the study of ferroptosis in relation to cisplatin resistance is still in its infancy, growing evidence suggests that inducing ferroptosis may help overcome chemotherapy resistance in various cancers (10, 11, 12, 13). For instance, ATF3 has been shown to reverse cisplatin resistance in gastric cancer by inducing ferroptosis *via* inhibition of the Nrf2/Keap1/xCT signaling pathway (10). Consequently, understanding how EOC cells evade cisplatin-induced ferroptosis could offer new therapeutic opportunities to combat platinum resistance.

p27, a member of the Cip/Kip family of cyclin-dependent kinase (CDK) inhibitors, is well-known for its role in regulating the cell cycle by inhibiting CDKs (14, 15). Beyond this canonical role, p27 has been implicated in various CDK-independent processes, including apoptosis, transcription, metabolism, and cell migration (16). Notably, p27 has been shown to influence multiple forms of cell death, including apoptosis and autophagy (17, 18). Although there is no direct evidence linking p27 to ferroptosis, p27's role in modulating intracellular redox homeostasis and influencing ROS production may indirectly affect tumor cell sensitivity to oxidative stress, potentially influencing ferroptosis (19, 20). Furthermore, studies have shown that p27 modulates chemotherapy response in various cancers, with its downregulation linked to poor prognosis and chemoresistance (21). However, its specific role in ferroptosis, particularly in the context of platinum resistance in EOC remains unclear.

In the present study, we demonstrated that low expression of p27 was associated with poor prognosis of EOC patients. Furthermore, functional assays confirmed that p27 deficiency played a pivotal role in mediating cisplatin resistance in EOC both *in vitro* and *in vivo*. Mechanistically, we found that p27 promoted ferroptosis by enhancing the transcriptional activity of cytochrome b-245 heavy chain (CYBB), a key positive regulator of ferroptosis, thereby increasing EOC cell sensitivity to cisplatin. CYBB, a component of the NADPH oxidase complex, contributes to ferroptosis by promoting ROS generation and lipid peroxidation (22). Importantly, in both *in vitro* and *in vivo* models, we demonstrated that a Skp2 inhibitor, which enhances p27 expression, promoted ferroptosis and restored EOC cell sensitivity to cisplatin, improving therapeutic efficacy. Our findings underscore the critical role of p27 in mediating ferroptosis, thereby sensitizing EOC cells to cisplatin therapy. These results highlight p27-based therapeutic strategies as a promising approach to overcoming EOC chemoresistance.

## Results

### p27 is associated with the prognosis and chemotherapy response of EOC patients

**Figure 1:**
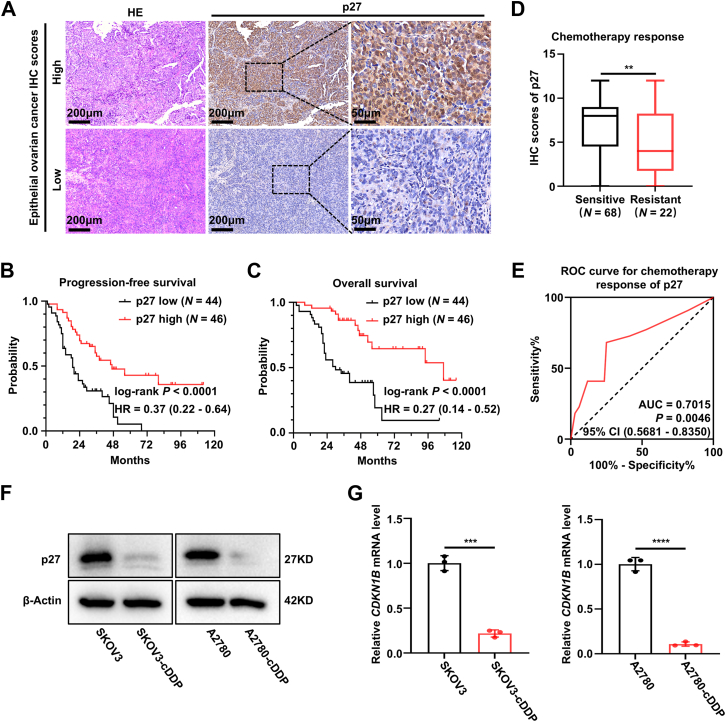
**p27 is associated with the prognosis and chemotherapy response of EOC patients**. *A*, representative IHC staining images of p27 in human epithelial ovarian cancer (EOC) tissues from 90 patients. This Scale bar represents 200 μm (100 × ), 50 μm (400 × ). *B* and *C*, Kaplan-Meier plots depicting progression-free survival (B) and overall survival (C) of EOC patients based on p27 expression (log-rank test). *D*, quantitative analysis of p27 expression in chemo-sensitive (*N* = 68) and chemo-resistant (*N* = 22) EOC tissues, based on IHC staining (unpaired two-tailed Student’s *t* test). *E*. receiver operating characteristic curve of p27 expression levels and chemotherapy response. *F*, Western blot analysis of p27 expression in parental and cisplatin-resistant SKOV3 and A2780 cells. β-Actin was used as a loading control. Three biologically independent experiments were performed. *G*, qRT-PCR analysis of *CDKN1B* (encoding p27) expression in parental and cisplatin-resistant SKOV3 and A2780 cells. *β-Actin* served as a loading control. Data are presented as mean ± SEM of three biologically independent experiments (unpaired two-tailed Student’s *t* test). ∗∗*p* < 0.01, ∗∗∗*p* < 0.001, ∗∗∗∗*p* < 0.0001. EOC, epithelial ovarian cancer; IHC, immunohistochemistry; qRT-PCR, quantitative real-time PCR.

To explore the clinical relevance of p27 in EOC, immunohistochemistry (IHC) was first conducted to assess p27 protein levels in 90 EOC tissue samples (patient clinical characteristics are summarized in Table 1). Kaplan-Meier survival analysis revealed that p27 expression positively correlates with both progression-free survival and overall survival in EOC patients (Fig. 1, *A*–*C*). Notably, we further examined the correlation between p27 expression and clinicopathological characteristics in EOC patients. Our analysis demonstrated that reduced p27 expression in EOC tissues was associated with increased chemoresistance (Table 1; Fig. 1*D*). Additionally, receiver operating characteristic curve analysis indicated that p27 can effectively classify chemotherapy responses (Fig. 1*E*). Furthermore, cisplatin-resistant EOC cell lines were established, and their increased IC_50_ values for cisplatin were validated using 3-(4,5-dimethylthiazol-2-yl)-2,5-diphenyltetrazolium bromide (MTT) assays (Fig. S1, *A* and *B*). Western blot and quantitative real-time PCR (qRT-PCR) analyses showed that p27 expression was lower in cisplatin-resistant EOC cell lines compared to parental EOC cell lines (Fig. 1, *F* and *G*). Taken together, these findings suggest the potential clinical utility of p27 as both a prognostic marker and predictor of chemotherapy response in EOC.

**Table 1:** Association between p27 expression to clinicopathological characteristics in ovarian cancer (*N* = 90)

Clinicopathological characteristics	Number of patients	p27 expression number (%)		
		Low	High	*P*
Age (years)^a^				0.547
<50	36	19 (21.1%)	17 (18.9%)	
≥50	54	25 (27.8%)	29 (32.2%)	
Histology				0.541
High-grade serous	68	32 (35.6%)	36 (40.0%)	
Others	22	12 (13.3%)	10 (11.1%)	
FIGO stage^b^				0.176
I-II	22	8 (8.9%)	14 (15.6%)	
III-IV	68	36 (40.0%)	32 (35.6%)	
Chemotherapy response^c^				0.010
Sensitive	68	28 (31.1%)	40 (44.4%)	
Resistant	22	16 (17.8%)	6 (6.7%)	

a Age at surgery.

b According to FIGO (International Federation of Gynaecology and Obstetrics) classification (2014).

c Chemotherapy resistant tumors were defined as those with relapse within 6 months after completing chemotherapy or progress during the primary chemotherapy.

### Depletion of p27 contributes to cisplatin resistance of EOC cells *in vitro* and *in vivo*

**Figure 2:**
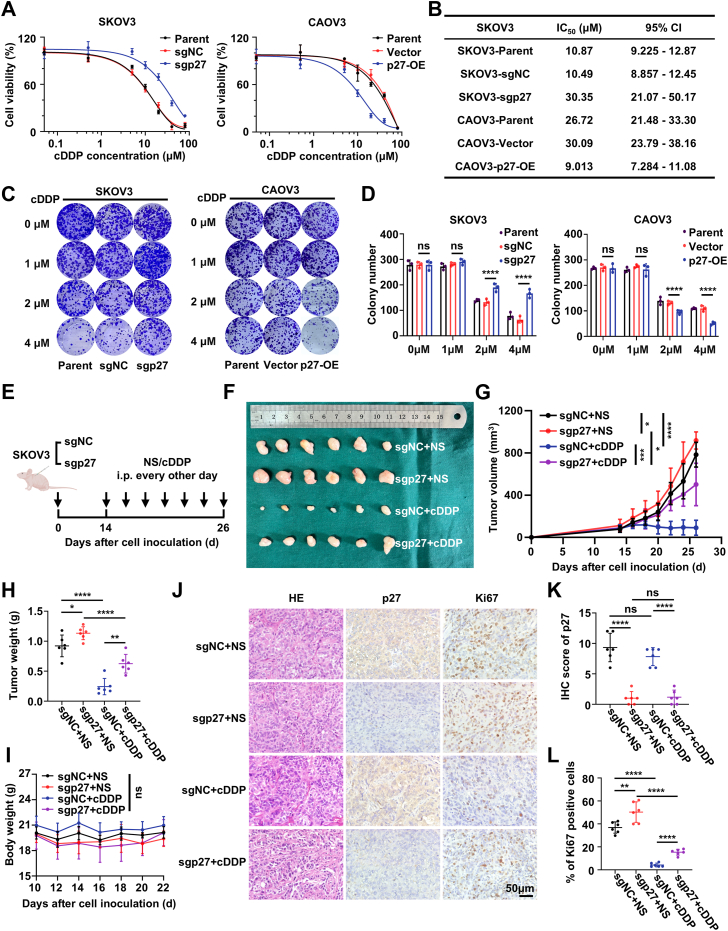
**Depletion of p27 contributes to cisplatin resistance of EOC cells *in vitro* and *in viv*o**. *A* and *B*, Cell viability (*A*) and IC_50_ values (*B*) of indicated EOC cells after treatment with various concentrations of cisplatin (cDDP) for 72 h. Data are presented as mean ± SEM of three biologically independent experiments. *C* and *D*, representative images (*C*) and quantification (*D*) of colony formation in the indicated EOC cells. Data represent mean ± SEM of three biologically independent experiments (two-way ANOVA). *E*, schematic diagram of the experimental design used to establish the animal model. *F*, images of excised tumors. *G* and *H*, tumor volume (*G*) and tumor weight (*H*) of sgNC/sgp27 SKOV3 subcutaneous xenografts in nude mice after treatment with NS or cDDP. Data are presented as mean ± SEM (n = 6 mice per group, two-way ANOVA). *I*, body weights of nude mice in the indicated groups. Data represent mean ± SEM (n = 6 mice per group, two-way ANOVA). *J*, representative IHC staining images of indicated proteins in tumor tissues from subcutaneous xenografts in nude mice. This scale bar represents 50 μm. *K* and *L*, quantification of IHC staining for p27 (K) and Ki67 (L) in tumor tissues from subcutaneous xenografts in nude mice. Data represent mean ± SEM of five random fields from six different mice (two-way ANOVA). ns: not significant, ∗*p* < 0.05, ∗∗*p* < 0.01, ∗∗∗*p* < 0.001, ∗∗∗∗*p* < 0.0001. EOC, epithelial ovarian cancer; IHC, immunohistochemistry.

To explore the role of p27 in cisplatin resistance in EOC, a series of *in vitro* experiments were conducted. The CRISPR/Cas9 system was used to knock down p27 in SKOV3 and A2780 cell lines (sgp27), while p27 overexpression was achieved in the CAOV3 cell line (p27-OE) *via* lentiviral transduction. The efficiency of p27 KO and overexpression was confirmed by Western blot and qRT-PCR (Fig. S2, *A* and *B*). Cell viability was assessed in p27-KO (sgp27) and p27-overexpressing (p27-OE) EOC cells treated with varying concentrations of cisplatin. sgp27 EOC cells exhibited increased cisplatin resistance, while p27-OE cells showed enhanced sensitivity compared to controls (Fig. 2, *A* and *B*; Fig. S2*C*). Colony formation assays were performed to evaluate the effect of p27 depletion or overexpression on EOC cells' response to cisplatin. The results indicated a significant increase in colony formation by sgp27 EOC cells in the presence of cisplatin, whereas p27-OE cells exhibited a marked decrease in colony formation compared to controls (Fig. 2, *C* and *D*; Fig. S2, *D* and *E*).

Next, we investigated the role of p27 in modulating cisplatin’s anti-tumor efficacy *in vivo*. To this end, sgNC/sgp27 SKOV3 cells were subcutaneously injected into 5-week-old female BALB/c nude mice. Starting on day 14, tumor-bearing mice were treated with either cisplatin (3 mg/kg) or saline every 2 days. Tumors were harvested for analysis on day 28 (Fig. 2*E*). p27 KO was found to promote tumor growth and reduce sensitivity to cisplatin (Fig. 2, *F*–*H*), without affecting mouse body weight (Fig. 2*I*). IHC staining of subcutaneous tumor tissues confirmed lower p27 expression and significantly increased Ki67 expression—a well-established marker of cell proliferation—in sgp27 tumors, regardless of cisplatin treatment, compared to sgNC tumors (Fig. 2, *J*–*L*).

To further confirm the specificity of this effect, re-expression of p27 in p27-KO cells (sgp27-p27-OE) restored cisplatin sensitivity, supporting that the change in cispaltin response is p27-dependent (Fig. S2, *F*–*H*).

### p27 is a key positive regulator of ferroptosis in EOC cells

**Figure 3:**
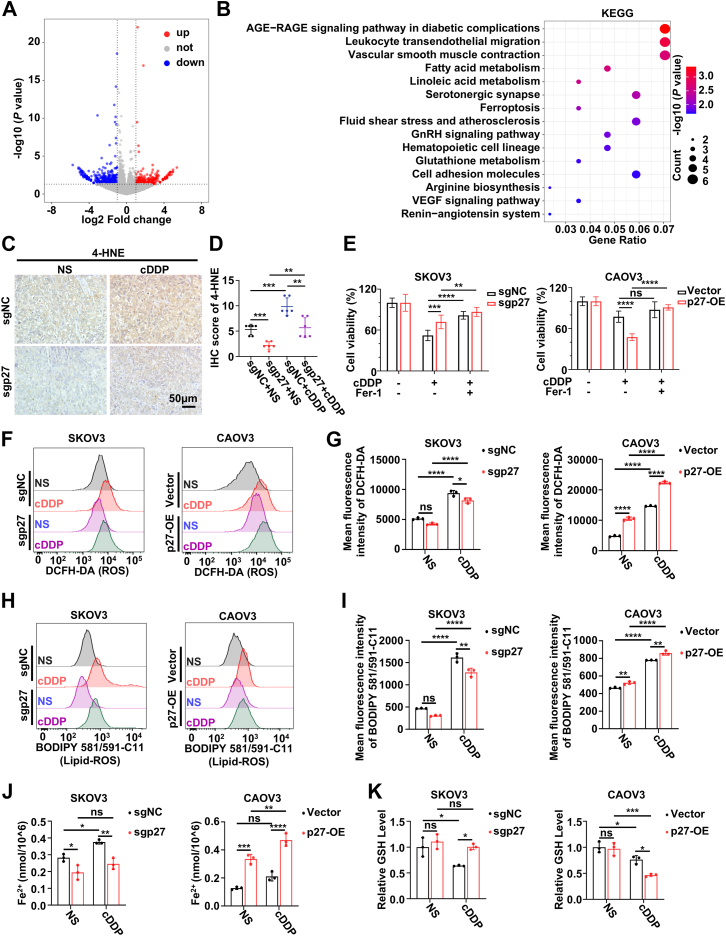
**p27 is a key positive regulator of ferroptosis in EOC cells**. *A*, volcano plot showing differentially expressed genes in sgp27-expressing *versus* sgNC-expressing SKOV3 cells. Red indicates upregulated genes (*p* < 0.05, Log FC > 1), and blue indicates downregulated genes (*p* < 0.05, Log FC < −1). *B*, KEGG enrichment analysis of differentially expressed genes in sgp27-expressing *versus* sgNC-expressing SKOV3 cells, showing the top 15 enriched pathways. *C* and *D*, representative images (*C*) and quantification (*D*) of IHC staining for 4-HNE in sgNC/sgp27-expressing SKOV3 subcutaneous xenografts in nude mice treated with NS or cDDP. Scale bar: 50 μm. Data represent mean ± SEM of five random fields from five different mice (two-way ANOVA). *E*, cell viability of indicated EOC cells treated with cDDP (20 μM) and ferrostatin-1 (10 μM) for 72 h. Data represent mean ± SEM of three biologically independent experiments (two-way ANOVA). *F* and *G*, representative images (*F*) and statistical analysis (*G*) of intracellular ROS in the indicated EOC cells treated with NS or cDDP (20 μM), stained with DCFH-DA, and measured by flow cytometry. Data represent mean ± SEM of three biologically independent experiments (two-way ANOVA). *H* and *I*, representative images (*H*) and statistical analysis (*I*) of intracellular lipid ROS in the indicated groups, stained with BODIPY 581/591-C11 and measured by flow cytometry. Data represent mean ± SEM of three biologically independent experiments (two-way ANOVA). *J* and *K*, Levels of Fe^2+^ (*J*) and GSH (*K*) in the indicated groups. Data represent mean ± SEM of three biologically independent experiments (two-way ANOVA). ns: not significant, ∗*p* < 0.05, ∗∗*p* < 0.01, ∗∗∗*p* < 0.001, ∗∗∗∗*p* < 0.0001. EOC, epithelial ovarian cancer; IHC, immunohistochemistry; ROS, reactive oxygen species.

To investigate the potential mechanism by which p27 depletion contributes to cisplatin resistance in EOC, RNA sequencing was performed on SKOV3-sgNC and SKOV3-sgp27 cells. Subsequent bioinformatics analysis identified 237 upregulated and 447 downregulated genes in the SKOV3-sgp27 group compared to the SKOV3-sgNC group (Fig. 3*A*). KEGG enrichment and GO analyses revealed that pathways such as fatty acid metabolism, ferroptosis, glutathione metabolism, and oxidoreductase activity were enriched, with the majority being related to ferroptosis (Fig. 3*B*; Fig. S3). Ferroptosis, first proposed by Scott J. Dixon *et al*., is a regulated form of cell death driven by iron-dependent lipid peroxidation (23). Recent studies have demonstrated that inducing ferroptosis can enhance cisplatin sensitivity in gastric, lung, and oral squamous cell carcinomas (10, 24, 25). IHC staining was performed on previously cisplatin-treated p27 KO subcutaneous tumor tissues to assess 4-HNE expression, a marker of ferroptosis. 4-HNE expression was found to be reduced in the p27 KO group compared to controls (Fig. 3, *C* and *D*). Therefore, we hypothesized that p27 depletion promotes cisplatin resistance in EOC by inhibiting ferroptosis.

To validate this hypothesis, MTT assays were performed to assess changes in cell viability following various treatments. Cisplatin-induced ferroptosis was reduced in sgp27 EOC cells compared to controls, but this effect was blocked by ferrostatin-1, a ferroptosis inhibitor. Conversely, p27-OE EOC cells exhibited increased ferroptosis (Fig. 3*E*; Fig. S4*A*). Flow cytometry analysis revealed no significant changes in apoptosis levels in either the sgp27 or p27-OE groups compared to controls, regardless of cisplatin treatment (Fig. S4, *B* and *C*). Consistently, IHC analysis of xenograft tumors showed that cleaved caspase-3 expression remained unchanged, further indicating that p27 regulates cisplatin sensitivity primarily through ferroptosis rather than apoptosis (Fig. S4*D*). Given that intracellular redox imbalance is a key driver of ferroptosis (26), we next examined oxidative stress–related parameters. ROS levels were detected using DCFH-DA, lipid-ROS was measured using BODIPY 581/591-C11, and Fe^2+^ levels were assessed using a Fe^2+^ detection kit in sgp27 and p27-OE groups following cisplatin treatment. Compared to controls, ROS, lipid-ROS, and Fe^2+^ levels were reduced in sgp27 EOC cells and elevated in p27-OE cells (Fig. 3, *F*–*J*; Fig. S4, *E*–*G*). Next, GSH levels were measured using a GSH assay kit, as GSH accumulation leads to ROS scavenging and ferroptosis inhibition (27). The results indicated significantly higher GSH levels in sgp27 EOC cells compared to controls, while p27-OE cells exhibited lower GSH levels (Fig. 3*K*; Fig. S4*H*). Consistently, re-expression of p27 in KO cells rescued the ferroptosis phenotype, as indicated by increased lipid ROS levels (Fig. S4*I*).

In addition to its role in ferroptosis, p27 is a critical negative regulator of the cell cycle. Therefore, we also examined the impact of p27 depletion on cell proliferation. p27 KO significantly promoted proliferation in EOC cells (Fig. 2*L*; Fig. S4*J*). This increase in proliferation could further contribute to the observed cisplatin resistance, as enhanced cell proliferation may reduce the effectiveness of the cisplatin treatment. In conclusion, p27 functions as a key positive regulator of ferroptosis in EOC cells, while also playing a crucial role in regulating cell proliferation.

### Depletion of p27 reduces Erastin-induced ferroptosis and attenuates its antitumor efficacy

**Figure 4:**
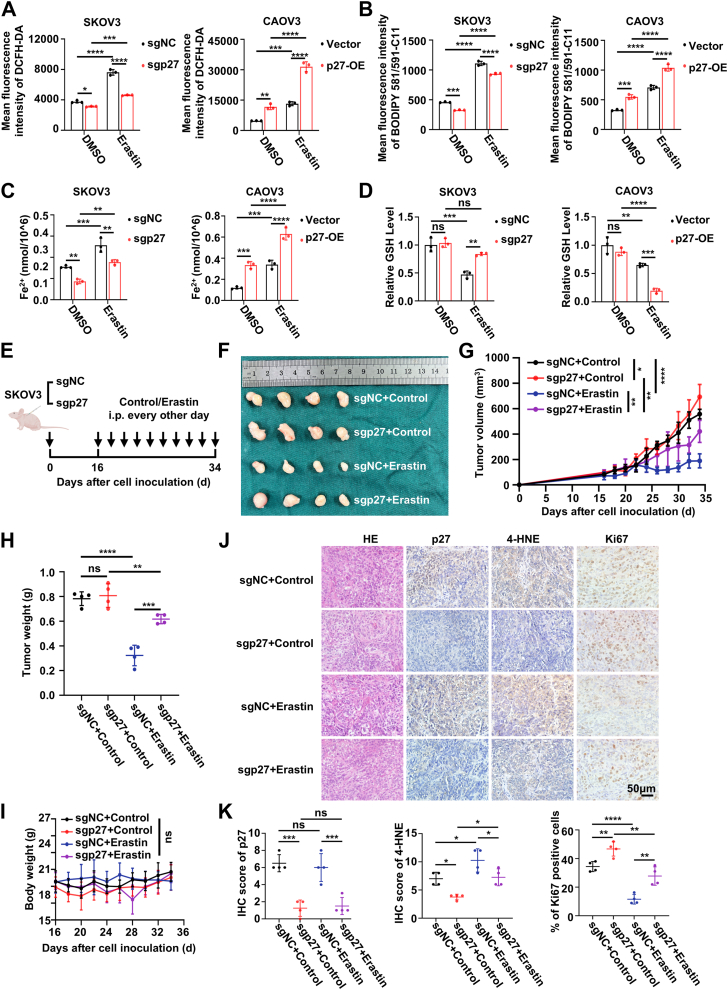
**Depletion of p27 reduces Erastin-induced ferroptosis and attenuates its antitumor efficacy**. *A* and *D*, levels of intracellular ROS (*A*), lipid ROS (*B*), Fe^2+^ (*C*), and GSH (*D*) in the indicated groups. Data represent mean ± SEM of three biologically independent experiments (two-way ANOVA). *E*, schematic diagram of the experimental design used to establish the animal model. *F*, images of removed tumors. *G* and *H*, tumor volume (*G*) and tumor weight (*H*) of sgNC/sgp27 SKOV3 subcutaneous xenografts in nude mice following solvent control or Erastin treatment. Data represent mean ± SEM (n = 4 mice per group, two-way ANOVA). *I*, body weights of nude mice in the indicated groups. Data represent mean ± SEM (n = 4 mice per group, two-way ANOVA). *J*, representative images of IHC staining of indicated proteins in tumor tissues from subcutaneous xenografts in nude mice. This scale bar represents 50 μm. *K*, quantification of IHC staining of p27, 4-HNE, and Ki67 in tumor tissues from subcutaneous xenografts in nude mice. Data represent mean ± SEM of five random fields of view from six different mice (two-way ANOVA). ns: not significant, ∗*p* < 0.05, ∗∗*p* < 0.01, ∗∗∗*p* < 0.001, ∗∗∗∗*p* < 0.0001. EOC, epithelial ovarian cancer; IHC, immunohistochemistry; ROS, reactive oxygen species.

Recent studies have demonstrated that Erastin, a known ferroptosis inducer, plays a beneficial role in antitumor activity (28). Upon Erastin treatment, ROS, lipid-ROS, and Fe^2+^ levels were reduced in sgp27 EOC cells and elevated in p27-OE EOC cells compared to controls (Fig. 4, *A*–*C*; Fig. S5, *A*–*E*). Additionally, Erastin treatment increased GSH levels in sgp27 EOC cells, while GSH levels were reduced in p27-OE EOC cells compared to controls (Fig. 4*D*; Fig. S5*F*). These findings suggest that p27 plays a critical role in Erastin-induced ferroptosis.

We further investigated the role of p27 in triggering ferroptosis *in vivo*. For this, we subcutaneously injected sgNC/sgp27 SKOV3 cells into 5-weeks female BALB/c nude mice. Starting on day 16, tumor-bearing mice were treated with Erastin (20 mg/kg) or solvent control every 2 days. Tumors were harvested and analyzed on day 36 (Fig. 4*E*). p27 KO impaired Erastin’s tumor-suppressive effect (Fig. 4, *F*–*H*), and the drug did not affect mouse body weight (Fig. 4*I*). IHC staining of subcutaneous tumor tissues further confirmed that Erastin treatment led to lower ferroptosis levels and higher proliferative capacity in the sgp27 group compared to the sgNC group (Fig. 4, *J* and *K*).

### p27 enhances the transcriptional activity of CYBB in EOC cells

**Figure 5:**
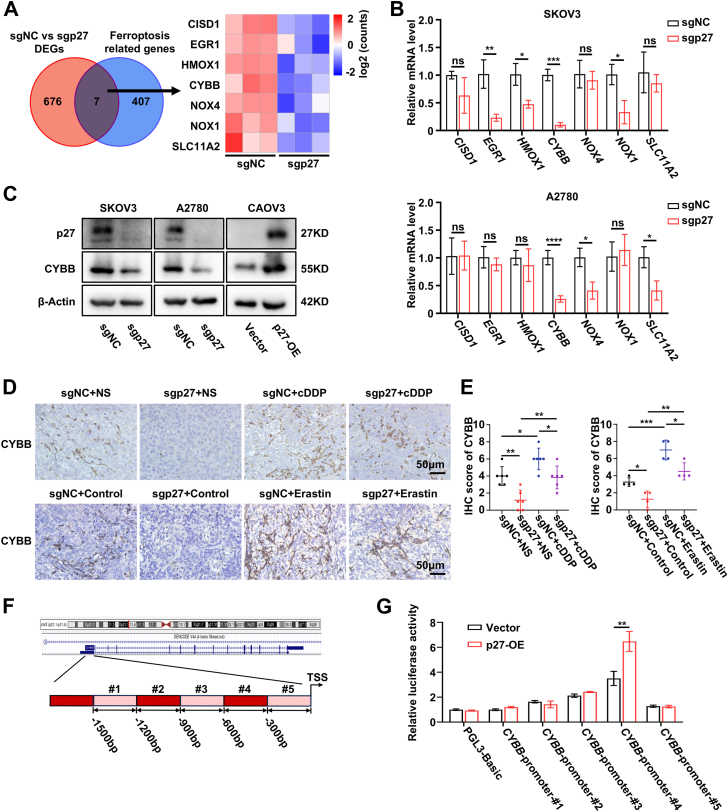
**p27 enhances the transcriptional activity of CYBB in EOC cells**. *A*, venn diagram showing the intersection of differentially expressed genes in sgp27 *versus* sgNC SKOV3 cells with ferroptosis-related genes from the FerrDb database (http://www.zhounan.org) and the heatmap showing seven ferroptosis-related genes among the differentially expressed genes. *B*, qRT-PCR analysis of indicated ferroptosis-related genes in indicated EOC cells. *β-Actin* was used as the loading control. Data represent mean ± SEM of three biologically independent experiments (unpaired two-tailed Student’s *t* test). *C*, Western blot analysis of p27 and CYBB expression in indicated EOC cells. β-Actin was used as the loading control. Three biologically independent experiments were performed. *D* and *E*, representative images (*D*) and quantification (*E*) of IHC staining of CYBB in sgNC/sgp27 SKOV3 subcutaneous xenografts in nude mice treated with NS, cDDP, or solvent control and Erastin. This scale bar represents 50 μm. Data represent mean ± SEM of five random fields of view per mouse (two-way ANOVA). *F*, schematic diagram of the different fragments of the CYBB promoter. *G*, relative luciferase activity of the CYBB promoter in p27-overexpressing CAOV3 cells. Data represent mean ± SEM of three biologically independent experiments (unpaired two-tailed Student’s *t* test). ∗*p* < 0.05, ∗∗*p* < 0.01, ∗∗∗*p* < 0.001, ∗∗∗∗*p* < 0.0001. CYBB, cytochrome b-245 heavy chain; EOC, epithelial ovarian cancer; IHC, immunohistochemistry.

To further explore the mechanism by which p27 regulates ferroptosis, we cross-referenced differentially expressed genes from RNA sequencing with ferroptosis-related genes from the FerrDb database. Visual analysis identified seven significantly downregulated ferroptosis-related genes in sgp27 SKOV3 cells (Fig. 5*A*). We then examined the mRNA levels of these genes in sgp27 SKOV3 and A2780 EOC cells and found that CYBB mRNA expression was significantly reduced (Fig. 5*B*). Western blot analysis revealed that CYBB protein levels were significantly reduced in p27-KO (sgp27) EOC cells and increased in p27-overexpressing (p27-OE) cells (Fig. 5*C*). Additionally, CYBB expression in cisplatin- and Erastin-treated subcutaneous tumor tissues was evaluated *via* IHC staining, which showed a positive correlation between p27 and CYBB expression (Fig. 5, *D* and *E*).

Increasing studies suggest that p27 might play an important role as a transcriptional regulator in tumor biological processes. Hence, we hypothesized that p27 may contribute to the expression of *CYBB* by enhancing its transcriptional activity. To test this hypothesis, we searched the NCBI database (https://www.ncbi.nlm.nih.gov) to predict the promoter sequence of *CYBB* and constructed luciferase reporter plasmids with different *CYBB* promoter regions which sequences were obtained from 1500 bp upstream of the transcription start site by uniformly truncating them into five segments. Dual-luciferase reporter assays were conducted in p27-OE CAOV3 cells transfected with five different CYBB promoter region plasmids or the PGL3-Basic control vector. The results indicated significantly increased CYBB-promoter-#4 activity (Fig. 5, *F* and *G*).

### p27 enhances cisplatin sensitivity in EOC by partially increasing CYBB expression and activating ferroptosis

**Figure 6:**
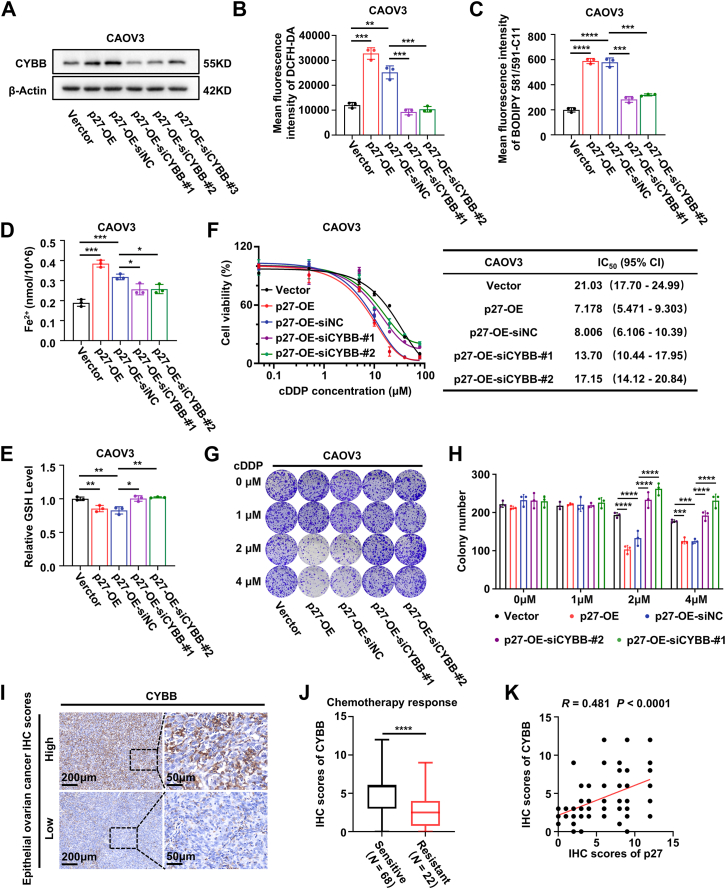
**p27 enhances cisplatin sensitivity in EOC by partially increasing CYBB expression and activating ferroptosis**. *A*, Western blot analysis of p27 expression in indicated EOC cells. β-Actin was used as the loading control. Three biologically independent experiments were performed. *B and E*, levels of intracellular ROS (*B*), lipid ROS (*C*), Fe^2+^ (*D*), and GSH (*E*) in the indicated groups. Data represent mean ± SEM of three biologically independent experiments (one-way ANOVA). *F*, cell viability and IC_50_ values of indicated EOC cells following treatment with cisplatin at various concentrations for 72 h. Data represent mean ± SEM of three biologically independent experiments. *G* and *H*, representative images (*G*) and quantification (*H*) of colony formation in indicated EOC cells. Data represent mean ± SEM of three biologically independent experiments (two-way ANOVA). *I*, representative IHC staining images of CYBB in human EOC tissues from 90 patients. The Scale bar represent: 200 μm (100 × ), 50 μm (400 × ). *J*, quantitative analysis of CYBB protein levels in EOC chemo-sensitive (*N* = 68) and chemo-resistant (*N* = 22) tissues based on IHC staining (unpaired two-tailed Student’s *t* test). *K*, correlation between the IHC scores of p27 and CYBB (Spearman’s correlation test). ∗*p* < 0.05, ∗∗*p* < 0.01, ∗∗∗*p* < 0.001, ∗∗∗∗*p* < 0.0001. EOC, epithelial ovarian cancer; ROS, reactive oxygen species.

Additional rescue experiments were performed to further validate the role of p27 in regulating CYBB-mediated ferroptosis. p27-OE CAOV3 cells were transfected with siRNAs targeting CYBB, and Western blot analysis confirmed the knockdown efficiency (Fig. 6*A*). CYBB knockdown partially reversed the p27 overexpression-induced increase in ROS, lipid ROS, and Fe^2+^ levels, as well as the decrease in GSH levels, in EOC cells, indicating that p27’s ferroptosis-promoting effects were inhibited (Fig. 6, *B*–*E*; Fig. S6, *A* and *B*). Cell viability was assessed after treatment with varying concentrations of cisplatin using MTT assays. The results demonstrated that CYBB downregulation significantly increased the cisplatin IC_50_ in p27-OE CAOV3 cells (Fig. 6*F*). Consistently, the colony formation assay indicated that CYBB downregulation significantly increased the number of colonies formed in p27-OE CAOV3 cells treated with cisplatin (Fig. 6, *G* and *H*). Furthermore, Overexpression of CYBB in SKOV3-cDDP cells significantly reduced cisplatin resistance, restoring cisplatin sensitivity to a level comparable with parental cells (Fig. S6, *C* and *D*).

Correspondingly, IHC staining revealed that CYBB expression was lower in chemo-resistant EOC tissues compared to chemo-sensitive tissues (Fig. 6, *I* and *J*). Importantly, a positive correlation was observed between CYBB and p27 expression levels in EOC tissues (R = 0.481, *p* < 0.0001) (Fig. 6*K*). Kaplan-Meier survival analysis revealed a positive correlation between CYBB expression and both PFS and OS in EOC patients (Fig. S7). Collectively, these findings suggest that p27 plays a critical role in maintaining intracellular redox homeostasis, inducing ferroptosis, and enhancing cisplatin sensitivity in EOC by upregulating CYBB expression.

### SKPin C1 increases the expression of p27 and enhances the anti-tumor effect of cisplatin of EOC *in vitro* and *in vivo*

**Figure 7:**
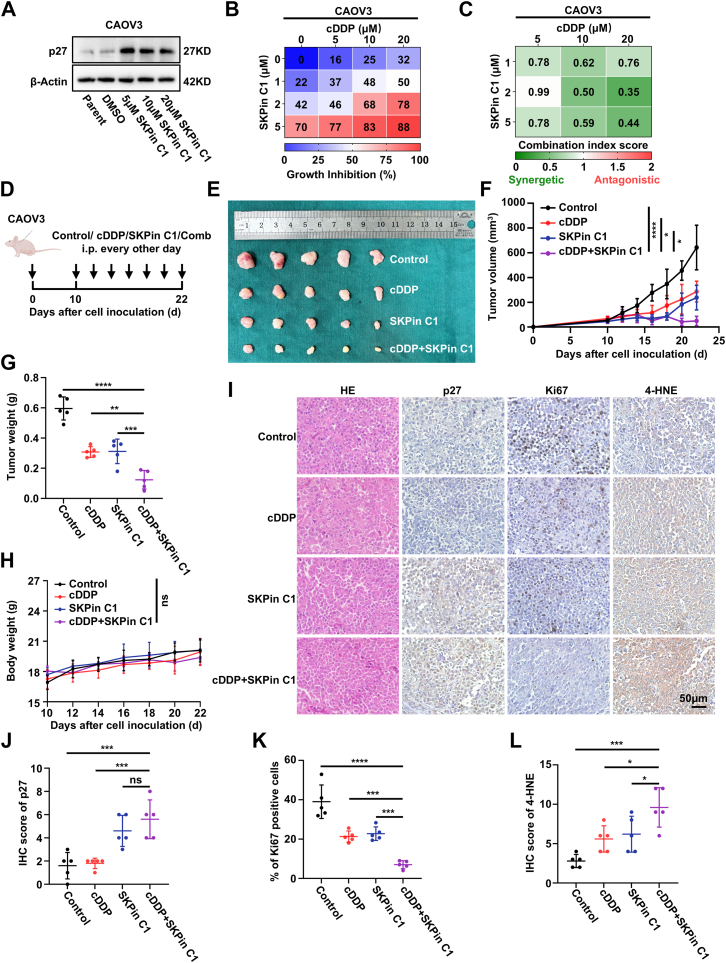
**SKPin C1 increases the expression of p27 and enhances the anti-tumor effect of cisplatin of EOC *in vitro* and *in viv*o**. *A*, Western blot analysis of p27 expression in CAOV3 cells following treatment with SKPin C1 at various concentrations for 24 h. β-Actin was used as the loading control. Three biologically independent experiments were performed. *B*, percentage inhibition at each concentration of cisplatin, SKPin C1, or their combination in CAOV3 cells. *C*, combination index (CI) scores for CAOV3 cells treated with cisplatin in combination with SKPin C1 at the indicated concentrations. *D*, schematic diagram of the experimental design used to establish the animal model. *E*, images of removed tumors. *F*–*G* Tumor volume (*F*) and tumor weight (*G*) of CAOV3 subcutaneous xenografts in nude mice following treatment with solvent control, cisplatin, SKPin C1, or cisplatin + SKPin C1. Data represent mean ± SEM (n = 5 mice per group, one-way ANOVA). *H*, body weights of nude mice in the indicated groups. Data represent mean ± SEM (n = 5 mice per group, one-way ANOVA). *I*, representative IHC staining images of indicated proteins in tumor tissues from subcutaneous xenografts in nude mice. This scale bar represent 50 μm. *J* and *L*, quantification of IHC staining for p27 (*J*), Ki67 (*K*), and 4-HNE (*L*) in tumor tissues from subcutaneous xenografts in nude mice. Data represent mean ± SEM of five random fields of view from five different mice (one-way ANOVA). ns: not significant, ∗*p* < 0.05, ∗∗*p* < 0.01, ∗∗∗*p* < 0.001, ∗∗∗∗*p* < 0.0001. CYBB, cytochrome b-245 heavy chain; CI, combination index; EOC, epithelial ovarian cancer; IHC, immunohistochemistry.

Given the lack of drugs specifically targeting p27, we sought to identify a compound that could inhibit p27 ubiquitination and prevent its degradation, thereby increasing its expression. SKPin C1, a highly selective small-molecule inhibitor of SKP2, targets the SKP2-mediated degradation of p27 (29). EOC cells were treated with varying concentrations of SKPin C1, and Western blot analysis revealed a significant increase in p27 protein levels following SKPin C1 treatment (Fig. 7*A*; Fig. S8*A*). SKPin C1 significantly induced ferroptosis in EOC cells (Fig. S8, *B*–*G*). Furthermore, MTT assays were performed to assess whether SKPin C1, in combination with cisplatin, enhanced antitumor efficacy in EOC cells. The results demonstrated that the combination of SKPin C1 with cisplatin exhibited stronger antitumor efficacy compared to cisplatin alone (Fig. 7, *B* and *C*; Fig. S8, *H* and *I*).

Based on the observed synergistic effect of SKPin C1 and cisplatin *in vitro*, we next investigated the antitumor efficacy of this combination *in vivo*. CAOV3 cells were subcutaneously injected into 5-week-old female BALB/c nude mice. Beginning on day 10, tumor-bearing mice were treated every 2 days with solvent control, cisplatin, SKPin C1, or the combination of cisplatin and SKPin C1. Tumors were harvested and analyzed on day 24 (Fig. 7*D*). The combination of cisplatin and SKPin C1 significantly inhibited the growth of EOC subcutaneous tumors (Fig. 7, *E*–*G*), with no significant differences in mouse body weight among the four groups (Fig. 7*H*). IHC staining of subcutaneous tumor tissues revealed increased expression of p27 and 4-HNE and decreased expression of Ki67 in the combination treatment group (Fig. 7, *I*–*L*). Collectively, our findings suggest that SKPin C1 may sensitize p27-deficient tumors to cisplatin treatment in EOC.

To confirm the specificity of Skpin C1, Skp2 was silenced by siRNAs in SKOV3 and CAOV3 cells. Skp2 knockdown similarly increased p27 levels, enhanced cisplatin sensitivity, and elevated lipid ROS, supporting that Skpin C1 exerts its effects through Skp2 inhibition (Fig. S9).

## Discussion

Our findings highlight the critical role of p27 in enhancing cisplatin sensitivity in EOC cells, primarily through its induction of ferroptosis. In this study, we demonstrated that p27 transcriptionally activates CYBB, resulting in elevated ROS levels and lipid peroxidation, which subsequently induces ferroptosis in EOC cells. These results provide compelling evidence that targeting p27 could represent an effective strategy for enhancing the sensitivity of EOC cells to platinum-based chemotherapy. Because baseline p27 levels differ markedly among EOC cell lines, we used high-p27 lines for KO and a low-p27 line for overexpression to ensure a meaningful biological response range.

Traditionally recognized as a cell cycle regulator that inhibits CDKs (14, 30), p27 has more recently been implicated in a variety of cellular processes, such as apoptosis, autophagy, and oxidative stress responses (17, 18, 19). Our study identifies an important role for p27 in regulating ferroptosis, an iron-dependent form of cell death driven by lipid peroxidation and ROS accumulation (23). Transcriptome sequencing revealed that p27 significantly influences pathways involved in ferroptosis, underscoring its regulatory role in this process. Ferroptosis has emerged as a promising therapeutic target in cancer, with studies indicating that it can overcome chemoresistance in ovarian, gastric, lung, and breast cancers (31, 32, 33). We found that p27 deficiency in EOC cells significantly reduced cisplatin-induced ferroptosis, as evidenced by lower ROS and lipid peroxidation levels, suggesting that cancer cells evade ferroptosis to develop chemoresistance. Furthermore, ferrostatin-1, a ferroptosis inhibitor, reversed cisplatin sensitivity in p27-overexpressing cells, underscoring the crucial role of ferroptosis in mediating cisplatin’s cytotoxic effects. These findings position p27 as a key factor in promoting ferroptosis, suggesting potential strategies to overcome chemoresistance in EOC.

Transcriptomic analysis identified CYBB, a ferroptosis-related gene significantly regulated by p27, as a member of the nicotinamide adenine dinucleotide phosphate (NADPH) oxidase (NOXs) family, an important source of intracellular ROS and a potential trigger of ferroptosis (34). As the catalytic subunit of the NOX2 complex, CYBB transfers electrons from NADPH to molecular oxygen, producing superoxide and downstream ROS such as hydrogen peroxide. These reactive oxygen species promote lipid peroxidation and oxidative stress, which are central biochemical events in ferroptosis (35). Downregulation or inactivation of CYBB in ovarian cancer cells has been shown to inhibit ferroptosis, thereby effectively promoting chemoresistance (36). Based on these studies, we hypothesized that CYBB could be a key downstream mediator in p27-regulated ferroptosis. We subsequently verified that p27 KO decreased CYBB mRNA and protein levels, while p27 overexpression increased CYBB expression. A series of rescue experiments demonstrated that CYBB downregulation in p27-overexpressing EOC cells partially reversed p27-induced ferroptosis and chemotherapy sensitization. These results provide further evidence that p27 deficiency contributes to EOC chemoresistance by inhibiting CYBB and ferroptosis. Moreover, new roles for p27 in transcriptional regulation are continuously being uncovered (37, 38). Previous studies have shown that p27 inhibits calcineurin 1 transcription, which in turn prevents Hsp90 degradation, stabilizes PHLPP1 protein, and mediates autophagy (39). It is hypothesized that p27 may act as a transcriptional regulator, modulating ferroptosis *via* the transcriptional activation of CYBB. We confirmed that p27 transcriptionally activates CYBB *via* luciferase reporter assays. The precise molecular mechanisms by which p27 regulates CYBB transcription need to be further elucidated. p27 may interact with specific transcription factors or co-factors to regulate CYBB expression, though this requires further investigation. Although our study focused on CYBB as a major downstream effector of p27, we cannot exclude the possibility that other ferroptosis regulators, such as GPX4 or SLC7A11, may also be indirectly influenced by p27, which warrants further investigation.

One of the most promising aspects of our findings is the therapeutic potential of targeting p27 to enhance cisplatin efficacy, especially through its role in promoting ferroptosis. Although there are currently no small-molecule drugs that specifically target p27 to increase its expression, we utilized a small-molecule inhibitor of Skp2 (SKPin C1), which selectively targets SCF-Skp2 activity by binding to the Skp2-p27 interface. This inhibitor specifically binds to the molecular surface pocket of the Skp2-Cks1 interface, preventing Skp2-mediated p27 ubiquitination while leaving the non-Skp2-p27 interface of active SCF unaffected (29). Our study demonstrated that combining cisplatin with SKPin C1 significantly enhanced tumor suppression and increased ferroptosis markers in EOC models. We observed that stabilized p27 leads to elevated ROS production and lipid peroxidation, both key drivers of ferroptosis. Notably, our results suggest that this approach may be especially effective in tumors with low baseline p27 expression, as p27-deficient EOC cells exhibited significantly increased sensitivity to cisplatin when treated with SKPin C1. This finding underscores the potential of Skp2 inhibitors to selectively target chemoresistant cancer cells, offering a dual benefit of enhancing ferroptosis and improving cisplatin efficacy. Collectively, our findings support the notion that combining Skp2 inhibition with cisplatin represents a promising therapeutic strategy to overcome cisplatin resistance, particularly in patients with low p27 expression, paving the way for more effective, personalized therapies in EOC. Although our *in vivo* models were limited to p27 KO and overexpression xenografts, complementary *in vitro* rescue and Skp2 knockdown experiments strongly support the mechanistic role of the Skp2-p27 axis in mediating ferroptosis and cisplatin sensitivity. Future studies incorporating *in vivo* rescue or Skp2-deficient models will further validate this pathway.

In conclusion, our study found that low p27 expression in ovarian cancer tissues correlates with chemoresistance, and that p27 overexpression triggers ferroptosis by transcriptionally activating CYBB, thereby enhancing cisplatin sensitivity in EOC cells. These findings offer potential avenues for developing new therapies to overcome cisplatin resistance in ovarian cancer.

## Experimental procedures

### Patients and samples

Ninety tissue samples were collected from patients diagnosed with epithelial ovarian cancer who underwent surgery followed by adjuvant platinum-based chemotherapy at Union Hospital, Tongji Medical College, Huazhong University of Science and Technology, between 2010 and 2017. Clinicopathologic characteristics were recorded, including age and time of surgery, histologic subtype, FIGO stage, and response to chemotherapy. Chemotherapy resistance was defined as relapse within 6 months of completing chemotherapy or disease progression during the initial chemotherapy regimen. The study protocol was approved by the Medical Ethics Committee of Union Hospital, Tongji Medical College, Huazhong University of Science and Technology, and all procedures involving human participants were conducted in accordance with the principles of the Declaration of Helsinki.

### IHC and quantification

Formalin-fixed, paraffin-embedded tumor sections from EOC patients and nude mice were deparaffinized, antigens retrieved, and endogenous peroxidase blocked using hydrogen peroxide solution. Sections were incubated overnight at 4 °C with the appropriate primary antibody. The next day, sections were incubated with secondary antibodies at 37 °C for 20 min, followed by 3,3′-diaminobenzidine and hematoxylin staining. IHC scores were calculated by multiplying staining intensity (0: negative; 1: weak; 2: moderate; 3: strong) by staining area (0: 0%; 1: < 25%; 2: 25–50%; 3: 50–75%; 4: > 75%). The nuclear IHC score was calculated by averaging the proportion of positive cells across five randomly selected 200 × fields.

### Cell culture and transfection

SKOV3 and CAOV3 cells were obtained from the China Center for Type Culture Collection, while A2780 cells were sourced from BLUEFBIO. All cell lines were authenticated *via* short tandem repeat profiling (Shanghai Biowing Applied Biotechnology). Parental EOC cells were treated with increasing concentrations of cisplatin for 12 cycles to generate cisplatin-resistant cell lines (SKOV3-cDDP and A2780-cDDP) (40). All cells were cultured in DMEM/F12·medium with 10% fetal bovine serum. Cisplatin-resistant cell lines were maintained in culture with 0.5 μg/ml cisplatin. All cells were cultured at 37 °C in a humidified incubator with·5% CO_2_ (Thermo Fisher Scientific).

The CRISPR/Cas9 system was used to construct p27-KO SKOV3 and A2780 cell lines using single guide RNA (sgRNA) (GeneChem,). The sequence of p27-sgRNA was 5′-AAGAGGCGAGCCAGCGCAAG-3′. A p27-overexpressing lentivirus (GeneChem) was used to construct the p27-overexpressing CAOV3 cell line. For transient knockdown, CYBB and Skp2 small interfering RNAs (siRNAs) (TsingKe) were transfected into EOC cells using Lipofectamine 3000 (Invitrogen, USA) following the manufacturer’s protocol. The target sequences of CYBB siRNAs were as follows: 5′-CCACCAAUCUGAAGCUCAA-3′(siRNA-#1), 5′-GGAAUCUCACCUUUCAUAA-3′(siRNA-#2) and 5′-GGCUGUGCAUAAUAUAACA-3′(siRNA-#3). The target sequences of Skp2 siRNAs were as follows: 5′-GAGCAAAGGGAGUGACAAATT UUUGUCACUCCCUUUGCUCTT-3′(siRNA-#1), 5′-CCAACCAUUGGCUGAACAUTTAUGUUCAGCCAAUGGUUGGTT-3′(siRNA-#2) and 5′-GAUAGUGUCAUGCUAAAGATTUCUUUAGCAUGACACUAUCTT-3′(siRNA-#3).

### Reagents and antibodies

Cisplatin was purchased from the Department of Pharmacy, Wuhan Union Hospital. Erastin (S7242), ferrostatin-1 (S7243), and Skp2 inhibitor C1 (SKPin C1) (S8652) were purchased from Selleck Chemical.

The primary antibodies used for IHC included anti-p27 (Proteintech; 25614-1-AP; 1:50 dilution), anti-Ki67 (Proteintech; 27309-1-AP; 1:3000 dilution), anti-4-HNE (R&D Systems; MAB3249; 1:200 dilution), anti-CYBB (Proteintech; 19013-1-AP; 1:100 dilution), and anti- Cleaved Caspase-3 (CST; 9661; 1:100 dilution). The primary antibodies used for Western blot included anti-p27 (Proteintech; 25614-1-AP; 1:1000 dilution), anti-CYBB (Proteintech; 19013-1-AP; 1:1000 dilution), anti-Skp2 (Proteintech; 15010-1-AP; 1:1000 dilution), anti-β-actin (ABclonal; AC026; 1:10,000 dilution), and anti-Vinculin (Proteintech; 26520-1-AP; 1:10,000 dilution).

### Western blot analysis (WB)

Total proteins were extracted using RIPA lysis buffer (Beyotime), separated *via* SDS-PAGE, and transferred to PVDF membranes (Millipore). Membranes were blocked with 5% skimmed milk for 2 h at room temperature and incubated overnight at 4 °C with the primary antibody. The next day, the membrane was incubated with secondary antibodies for 2 h at room temperature, and then signals were detected using ECL detection reagents.

### RNA extraction and quantitative real-time PCR (qRT-PCR)

Total RNA was extracted with Trizol lysis buffer (Thermo Fisher Scientific), and 1 μg of RNA was reverse transcribed into cDNA using HiScript III All-in-one RT SuperMix for qPCR (Vazyme). Subsequently, qRT-PCR was conducted using AceQ qPCR SYBR Green Master Mix (Vazyme) on a Step-One Plus Real-Time PCR system. *β-ACTIN* was used as the reference gene. The relative expression levels of mRNA were normalized using the 2^-ΔΔCt^ method (41). The primers used for qRT-PCR assays are listed in Supporting Table S1.

### Cell viability assay

Cell viability was assessed using an MTT reagent (Aladdin). EOC cells were seeded in 96-well plates and incubated for 72 h with varying drug concentrations after adherence. Afterward, 20 μl of MTT solution (5 mg/ml) was added to 200 μl of fresh medium per well, followed by incubation for 4 h at 37 °C with 5% CO2. Then, 150 μl of dimethyl sulfoxide was added to each well and gently shaken for 15 min until the crystals dissolved. The absorbance at 570 nm was then measured using a microplate reader (SpectraMax). IC_50_ values were calculated with GraphPad Prism 9.4 software (https://www.graphpad.com/scientific-software/prism/). Synergistic effects between two compounds were evaluated using CompuSyn software (https://www.combosyn.com) to generate a Combination Index (CI), where CI < 1 indicates synergy and CI > 1 indicates antagonism.

### Colony formation assay

Cells (500 cells/well) were seeded in six-well plates, and following adherence, various concentrations of cisplatin were added. The cells were incubated for 1 to 2 weeks until colonies formed. Colonies were then fixed with methanol and stained with crystal violet. Colonies were photographed and quantified using ImageJ software (https://imagej.nih.gov/ij).

### RNA sequencing analysis

Briefly, total RNA was extracted from SKOV3 vector control and SKOV3 p27-KO cells, and RNA quality was assessed using the A260/A280 ratio with a Nanodrop (Thermo Fisher Scientific). RNA integrity was assessed using agarose gel electrophoresis. RNA sequencing was performed by Novogene. Differentially expressed genes between groups were analyzed using the DESeq2 software package (https://bioconductor.org/packages/release/bioc/html/DESeq2.html). A *p* value of 0.05 and a fold change rate of 1 were used as thresholds to determine the statistical significance of the detected genes. Differentially expressed genes were analyzed by Gene Ontology analysis and KEGG enrichment using RStudio software (https://www.rstudio.com). Venn diagrams of differentially expressed genes (sgp27 vs sgNC SKOV3) overlapping with ferroptosis-related genes in the FerrDb database (http://www.zhounan.org) and heatmaps of seven ferroptosis-related genes were generated using RStudio software.

### ROS and lipid ROS assay

Cells were collected using EDTA-free trypsin and washed three times with PBS. For ROS analysis, 200 μl of 10 μM DCFH-DA (S0033S, Beyotime) was added, and for lipid ROS analysis, 200 μl of 10 μM BODIPY-581/591 C11 (D3861, Thermo Fisher Scientific) was used. The samples were incubated at 37 °C with 5% CO2 for 30 min, washed three times, and resuspended in 200 μl PBS. Flow cytometry was performed (BD), and data were analyzed using FlowJo 10 software (https://www.flowjo.com).

### Fe^2+^ and GSH assay

After treatment, intracellular Fe^2+^ concentrations were measured using the Fe^2+^ Assay Kit (E-BC-K881-M, Elabscience), and intracellular GSH levels were quantified with the Reduced GSH Assay Kit (A006-2-1, Nanjing Jianchen Bioengineering Institute), following the manufacturer's protocols.

### Apoptosis analysis

Cells were collected with EDTA-free trypsin, washed twice with PBS, and resuspended in 100 μl of 1 × buffer. Five μl of PI and 5 μl of FITC were added to the samples, followed by a 30-min incubation at room temperature. Cells were analyzed by flow cytometry (BD, USA) and processed using FlowJo 10 software (BD).

### CCK-8 assay

The proliferation ability of EOC cells was evaluated using the CCK-8 reagent (Vazyme). EOC cells were seeded in 96-well plates, and the optical density values were measured at 24 h, 48 h, and 72 h 10 μl of CCK-8 reagent were added to each culture well and incubated for 2 h. Finally, the optical density values were read at a wavelength of 450 nm.

### Luciferase reporter gene assay

Promoter sequences of CYBB were predicted using the NCBI database (https://www.ncbi.nlm.nih.gov), and luciferase reporter plasmids were constructed containing different CYBB promoter regions obtained from 1500 bp upstream of the transcription start site, which was truncated into five segments. CAOV3 p27-OE cells were transfected with 200 ng of CYBB promoter plasmids or the PGL3-Basic plasmid as a negative control. Luciferase activity was analyzed by co-transfecting cells with 20 ng of Renilla plasmid and comparing firefly to Renilla luciferase activities. A dual-luciferase reporter assay system (E1910, Promega) was used to measure luciferase activity 48 h post-transfection.

### Animal studies

Three separate animal experiments were conducted in this study, each with distinct objectives (1). p27-KO and control SKOV3 cells (5 × 10^6^ cells) were injected subcutaneously into female BALB/c nude mice. When tumors reached 50 to 100 mm^3^, mice were randomly assigned to receive saline or cisplatin (3 mg/kg) every 2 days (2). p27-KO and control SKOV3 cells (5 × 10^6^ cells) were injected subcutaneously into female BALB/c nude mice. When tumors reached 50 to 100 mm^3^, mice were randomly assigned to receive solvent control or Erastin (20 mg/kg) every 2 days (3). CAOV3 cells (5 × 10^6^ cells) were injected subcutaneously into female BALB/c nude mice. When tumors reached 50 to 100 mm^3^, mice were randomly assigned to receive solvent control, cisplatin (3 mg/kg), SKPin C1 (10 mg/kg), or a combination of cisplatin and SKPin C1 every 2 days.

Tumor growth was monitored by measuring tumor diameters every 2 days, and tumor volume was calculated using the formula: 0.5 × length × width^2^. At the end of the treatment, tumors were excised, weighed, and embedded in tissue. All animal experiments were conducted at Wuhan Youdu Biotechnology Co., Ltd and were approved by the Institutional Animal Care and Use Committee of Wuhan Youdu Biotechnology Co., Ltd

### Statistical analysis

All experiments were conducted at least three times. Data are presented as mean ± SD. Statistical significance was determined using the appropriate tests in GraphPad Prism 9.4 software. Survival curves were generated using the Kaplan-Meier method and compared *via* the log-rank test. For all tests, *p* < 0.05 was considered statistically significant (ns: not significant, ∗*p* < 0.05, ∗∗*p* < 0.01, ∗∗∗*p* < 0.001, ∗∗∗∗*p* < 0.0001).

### Ethics approval and consent to participate

All experiments involving human samples and clinical data were approved by the Medical Ethics Committee of Union Hospital, Tongji Medical College, Huazhong University of Science and Technology. All animal experiments were conducted at Wuhan Youdu Biotechnology Co., Ltd and were approved by the Institutional Animal Care and Use Committee of Wuhan Youdu Biotechnology Co., Ltd

## Data availability

All data generated or analyzed during this study are available from the corresponding author on reasonable request.

## Supporting information

This article contains supporting information.

## Conflict of interest

The authors declare that they have no conflicts of interest with the contents of this article. Supporting Information
