# Supplementary material for: The cyclin-dependent kinase inhibitor p27 facilitates chemosensitivity by promoting ferroptosis in epithelial ovarian cancer
Source: J Biol Chem. 2025 Dec 5;302(1):111011. doi: 10.1016/j.jbc.2025.111011 (PMC12800695; doi:10.1016/j.jbc.2025.111011)
Supplement: Supplementary Material 1 [file mmc1.docx]

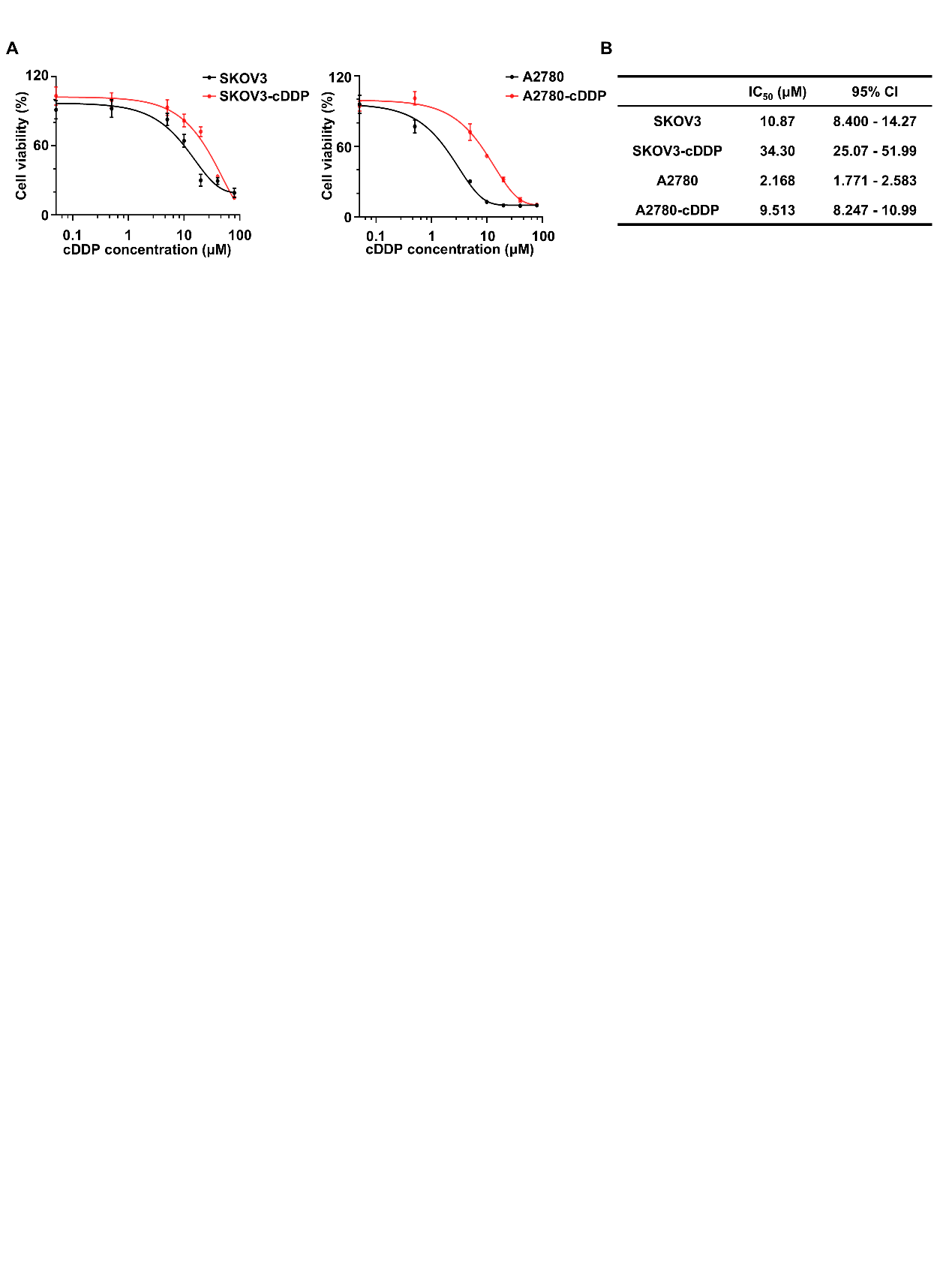
**Figure S1. The IC_50_ of cisplatin-resistant EOC cell lines detected by MTT assay.**

**A-B** Cell viability (A) and IC_50_ values (B) of parental and cisplatin-resistant SKOV3 and A2780 cells following treatment with cisplatin at various concentrations for 72 h. Data represent mean ± SEM of three biologically independent experiments.


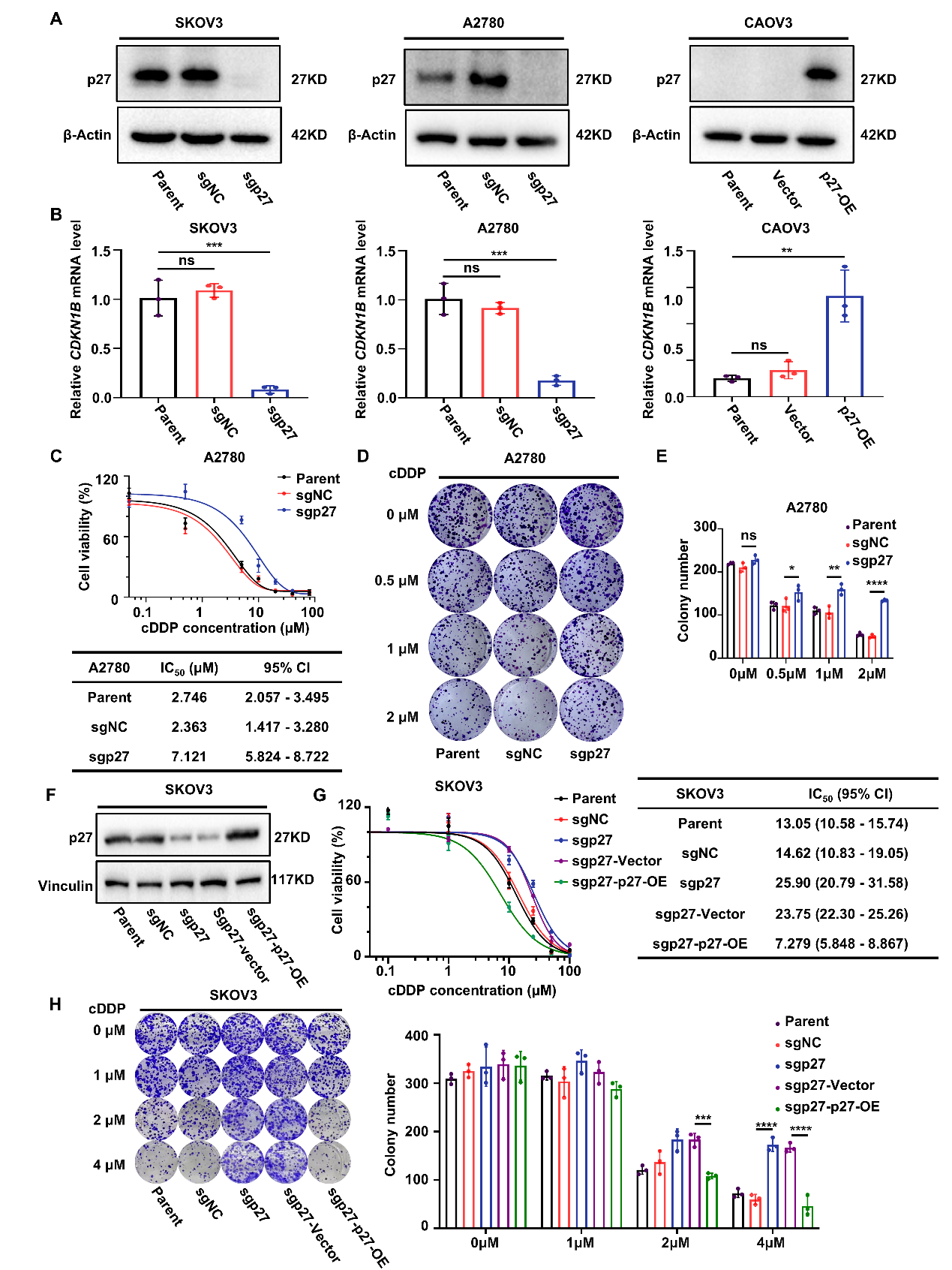
 **Figure S2. Depletion of p27 contributes to cisplatin resistance of EOC cells.**

**A** Western blot analysis of p27 expression in indicated EOC cells. β-Actin was used as the loading control. Three biologically independent experiments were performed. **B** qRT-PCR analysis of *CDKN1B* expression in indicated EOC cells. *β-Actin* was used as the loading control. Data represent mean ± SEM of three biologically independent experiments (one-way ANOVA). **C** Cell viability and IC_50_ values of indicated EOC cells following treatment with cisplatin at various concentrations for 72 h. Data represent mean ± SEM of three biologically independent experiments. **D-E** Representative images (D) and quantification (E) of colony formation in indicated EOC cells. Data represent mean ± SEM of three biologically independent experiments (two-way ANOVA). **F** Western blot analysis of p27 expression in indicated EOC cells. Vinculin was used as the loading control. Three biologically independent experiments were performed. **G** Cell viability and IC_50_ values of indicated EOC cells following treatment with cisplatin at various concentrations for 72 h. Data represent mean ± SEM of three biologically independent experiments. **H** Representative images and quantification of colony formation in indicated EOC cells. Data represent mean ± SEM of three biologically independent experiments (two-way ANOVA). ns: not significant, **P* < 0.05, ***P* < 0.01, ****P* < 0.001, *****P* < 0.0001.


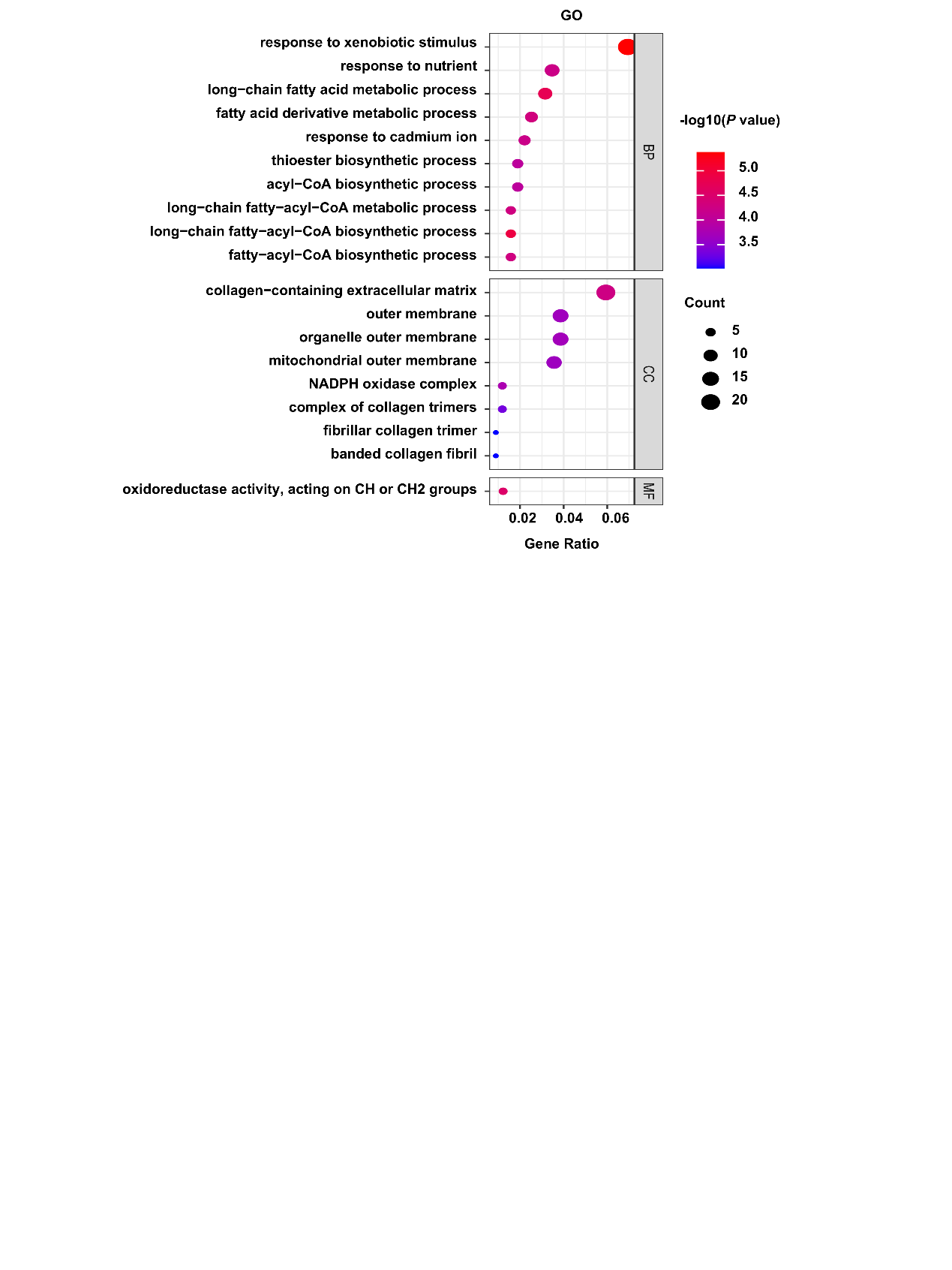
**Figure S3. Gene Ontology (GO) analysis in p27-deficient EOC cells.**

GO analysis of differentially expressed genes in sgp27-expressing versus sgNC-expressing SKOV3 cells showing 19 enriched pathways.


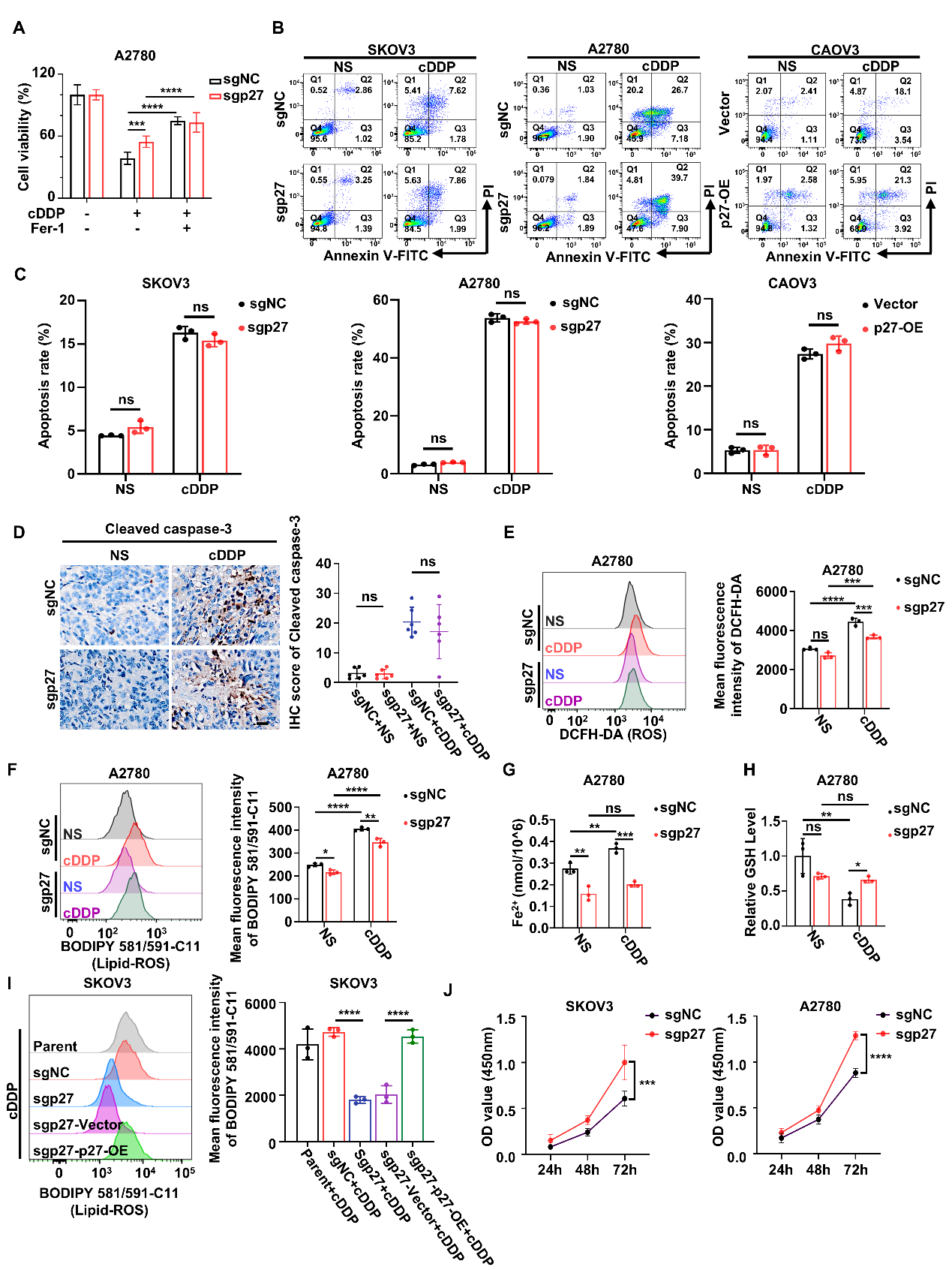


**Figure S4. Effects of p27 on cisplatin-induced apoptosis and ferroptosis in EOC cells.**

**A** Cell viability of indicated EOC cells following treatment with cisplatin (10 μM) and Fer-1 (10 μM) for 72 h. Data represent mean ± SEM of three biologically independent experiments (two-way ANOVA). **B-C** Representative FACS images (B) and statistical analysis (C) of apoptotic cells in indicated EOC cells treated with cisplatin (20 μM for SKOV3 and CAOV3, 10 μM for A2780) for 24 h. Data represent mean ± SEM of three biologically independent experiments (two-way ANOVA). **D** Representative images and quantification of IHC staining for Cleaved caspase-3 in tumor tissues from subcutaneous xenografts in nude mice. Data represent mean ± SEM of five random fields from six different mice (two-way ANOVA). **E** Representative images and statistical analysis showing intracellular ROS in the indicated groups. Data represent mean ± SEM of three biologically independent experiments (two-way ANOVA). **F** Representative images and statistical analysis showing intracellular lipid ROS in the indicated groups. Data represent mean ± SEM of three biologically independent experiments (two-way ANOVA). **G-H** Levels of Fe^2+^ (G) and GSH (H) in the indicated groups. Data represent mean ± SEM of three biologically independent experiments (two-way ANOVA). **I** Representative images and statistical analysis showing intracellular lipid ROS in the indicated groups. Data represent mean ± SEM of three biologically independent experiments (one-way ANOVA). **J** CCK-8 assay to detect the proliferative capacity of the cells after knocking down of the p27 by SKOV3 and A2780, respectively. Data represent mean ± SEM of three biologically independent experiments (two-way ANOVA). ns: not significant, **P* < 0.05, ***P* < 0.01, ****P* < 0.001, *****P* < 0.0001.


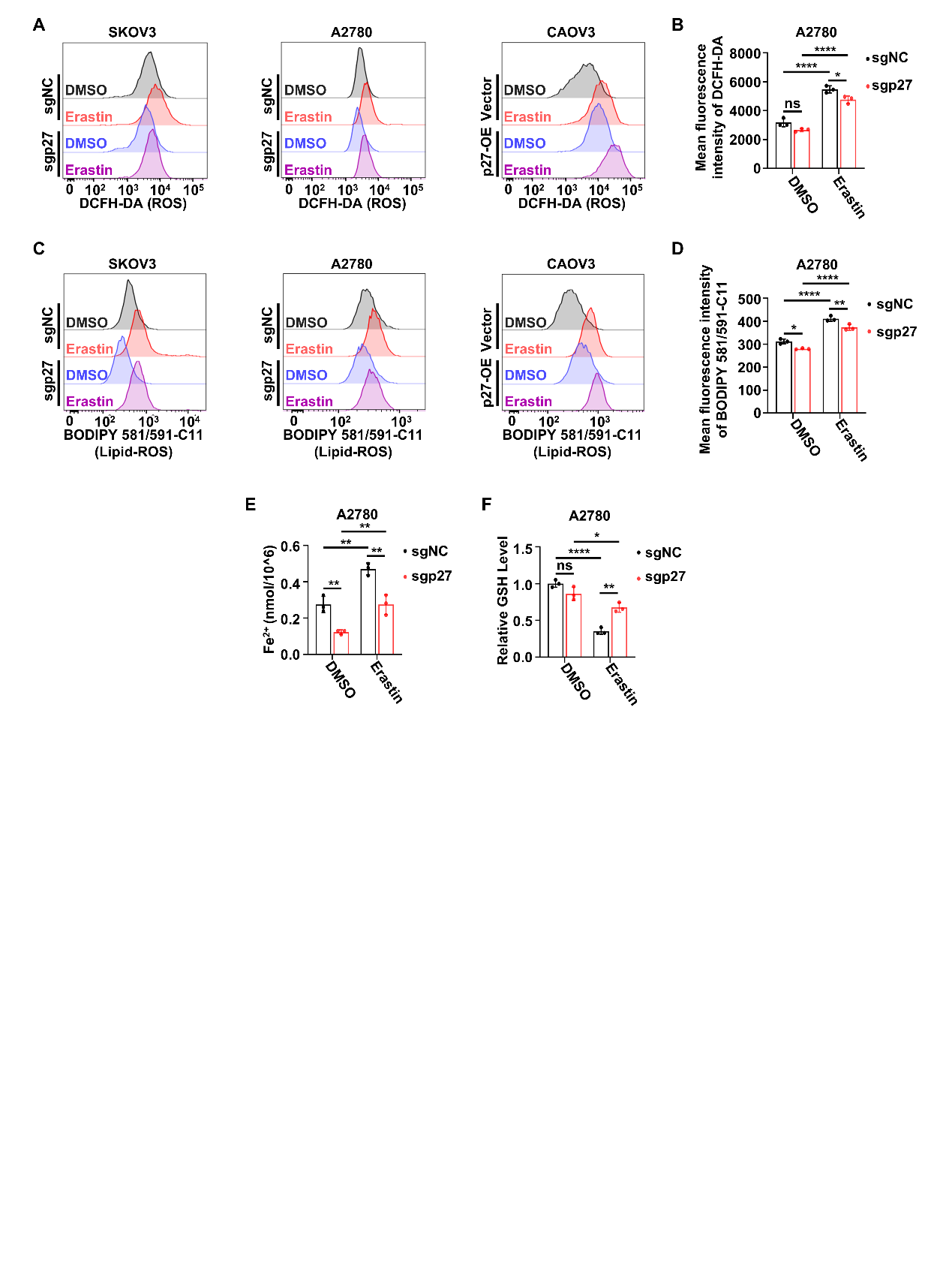
**Figure S5. Effects of p27 on Erastin-induced ferroptosis in EOC cells.**

**A-B** Representative images (A) and statistical analysis (B) showing intracellular ROS in the indicated groups. Data represent mean ± SEM of three biologically independent experiments (two-way ANOVA). **C-D** Representative images (C) and statistical analysis (D) showing intracellular lipid ROS in the indicated groups. Data represent mean ± SEM of three biologically independent experiments (two-way ANOVA). **E-F** Levels of Fe^2+^ (E) and GSH (F) in the indicated groups. Data represent mean ± SEM of three biologically independent experiments (two-way ANOVA). ns not significant, **P* < 0.05, ***P* < 0.01, ****P* < 0.001, *****P* < 0.0001.


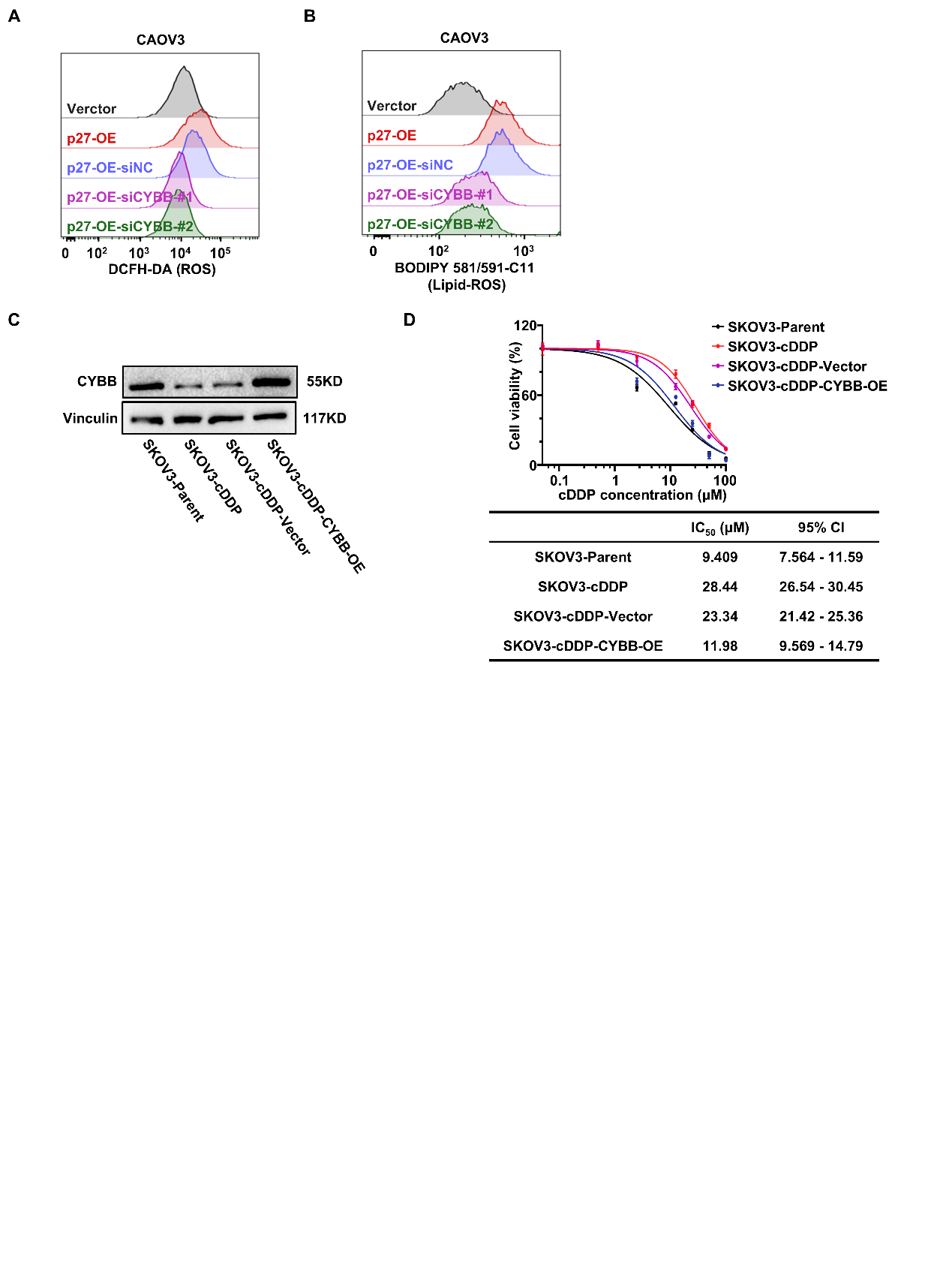


**Figure S6. p27 promotes ferroptosis of EOC cells by regulating CYBB expression.**

**A-B** Representative images showing intracellular ROS (A) and lipid ROS (B) in the indicated groups. Data represent mean ± SEM of three biologically independent experiments (one-way ANOVA). **C** Western blot analysis of p27 and CYBB expression in indicated EOC cells. Vinculin was used as the loading control. Three biologically independent experiments were performed. **D** Cell viability and IC_50_ values of indicated EOC cells following treatment with cisplatin at various concentrations for 72 h. Data represent mean ± SEM of three biologically independent experiments.


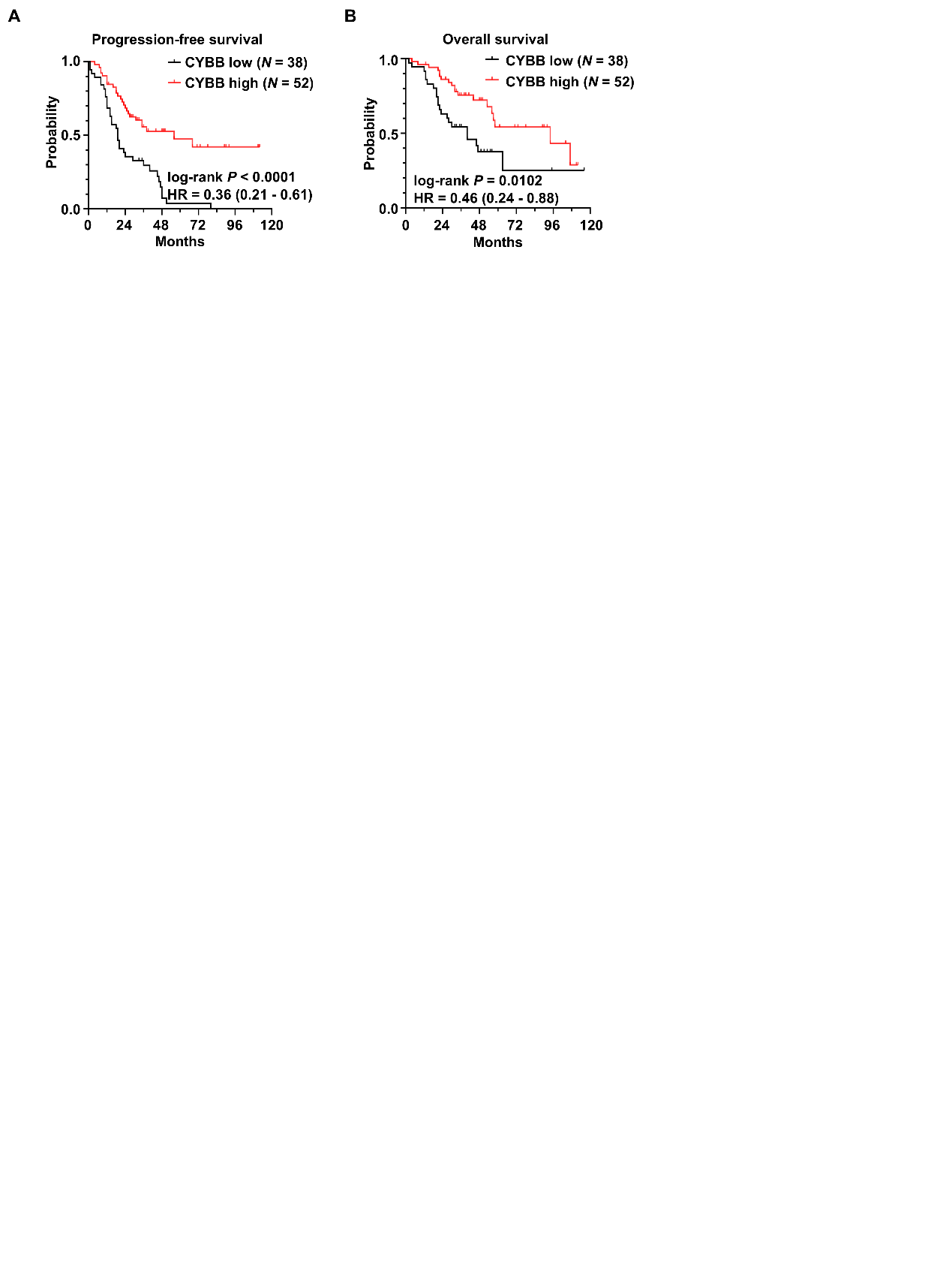
**Figure S7. CYBB expression is positively associated with progression-free survival and overall survival.**

**A-B** Kaplan-Meier plots depicting progression-free survival (A) or overall survival (B) of EOC patients based on CYBB expression (log-rank test).


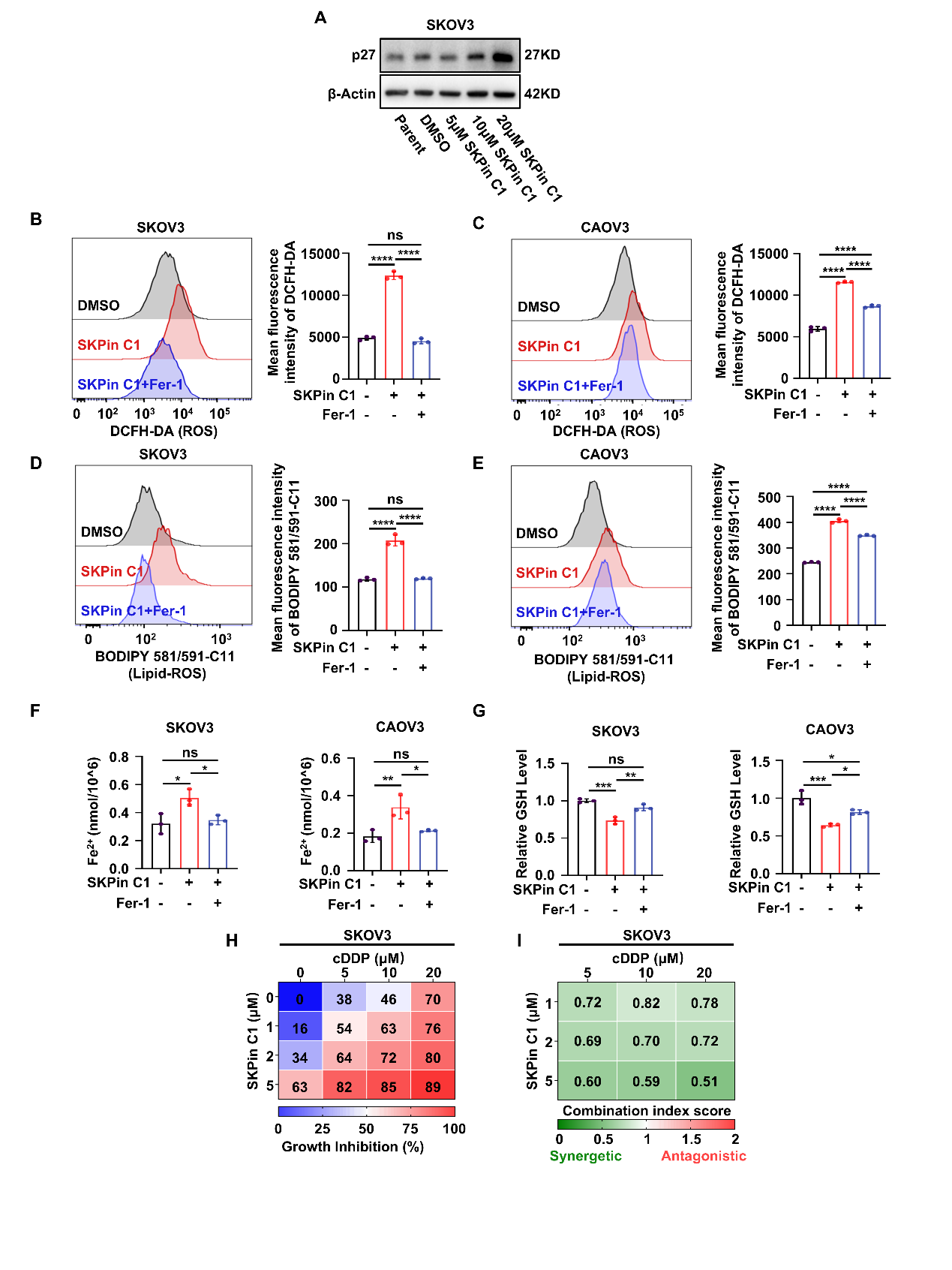
**Figure S8. SKPin C1 increases p27 expression and promotes EOC ferroptosis.**

**A** Western blot analysis of p27 expression in SKOV3 cells following treatment with SKPin C1 at various concentrations for 24 h. β-Actin was used as the loading control. Three biologically independent experiments were performed. **B-C** Representative images (B) and statistical analysis (C) showing intracellular ROS in SKOV3 and CAOV3 cells treated with solvent control, SKPin C1 (5 μM), or SKPin C1 + Fer-1 (10 μM). Data represent mean ± SEM of three biologically independent experiments (one-way ANOVA). **D-E** Representative images (D) and statistical analysis (E) showing intracellular lipid ROS in the indicated groups. Data represent mean ± SEM of three biologically independent experiments (one-way ANOVA). **F-G** Levels of Fe^2+^ (F) and GSH (G) in the indicated groups. Data represent mean ± SEM of three biologically independent experiments (one-way ANOVA). **H** Percentage inhibition at each concentration of cisplatin, SKPin C1, or their combination in CAOV3 cells. **I** Combination index (CI) scores for CAOV3 cells treated with cisplatin in combination with SKPin C1 at the indicated concentrations. ns not significant, **P* < 0.05, ***P* < 0.01, ****P* < 0.001, *****P* < 0.0001.


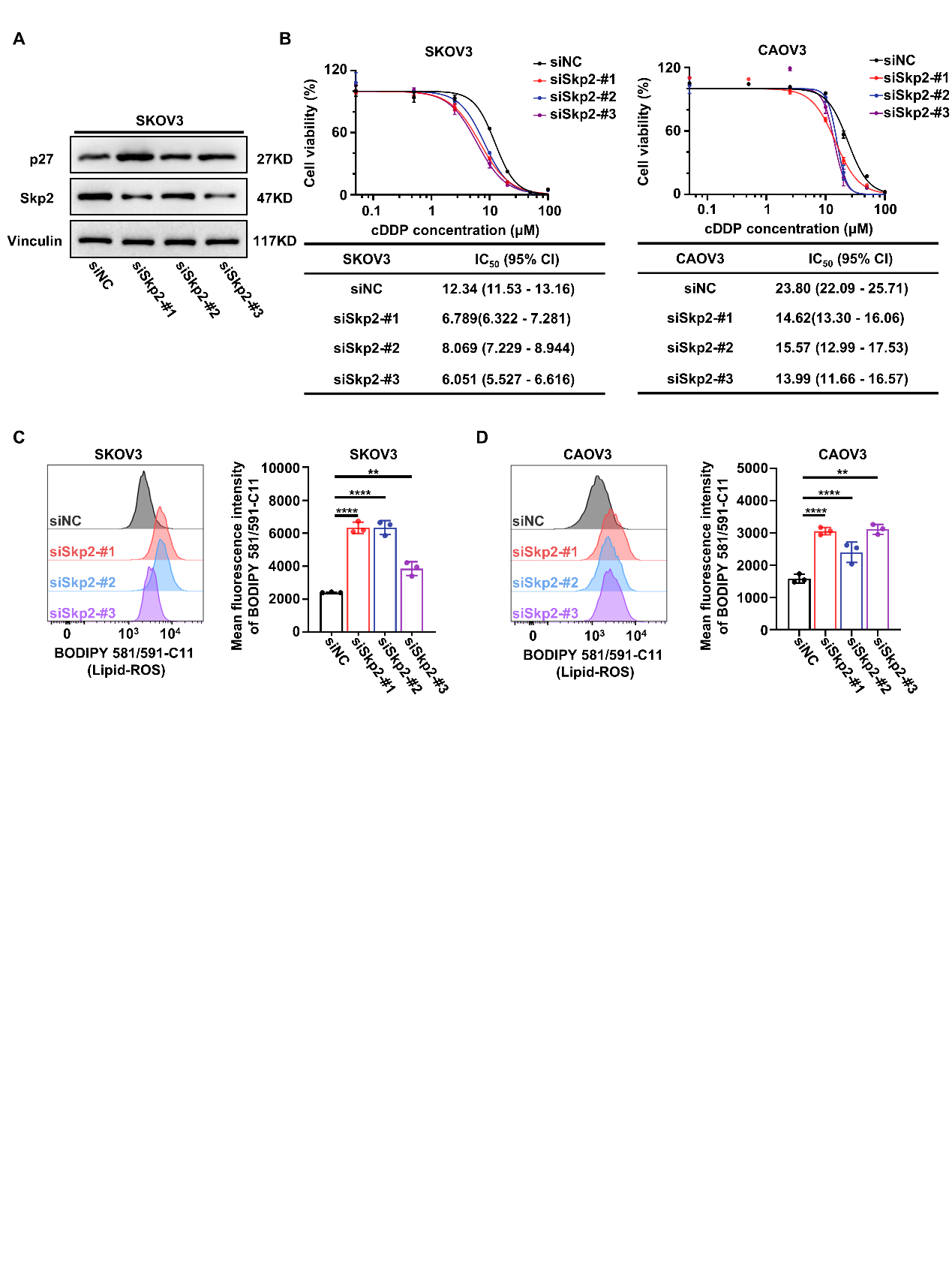


**Figure S9. Skp2 knockdown enhances cisplatin sensitivity and ferroptosis in EOC cells via upregulation of p27**

**A** Western blot analysis of p27 expression in indicated EOC cells. Vinculin was used as the loading control. Three biologically independent experiments were performed. **B** Cell viability and IC_50_ values of indicated EOC cells following treatment with cisplatin at various concentrations for 72 h. Data represent mean ± SEM of three biologically independent experiments. **C-D** Representative images and statistical analysis showing intracellular lipid ROS in the indicated groups of SKOV3 (C) and CAOV3 (D). Data represent mean ± SEM of three biologically independent experiments (one-way ANOVA). ns not significant, **P* < 0.05, ***P* < 0.01, ****P* < 0.001, *****P* < 0.0001.

| Supporting Table S1. The primers for qRT-PCR assays. | | |
| --- | --- | --- |
| Gene name | Primer | Primer Sequences |
| *CDKN1B* | Forward | 5'-AACGTGCGAGTGTCTAACGG-3' |
|  | Reverse | 5'-CCCTCTAGGGGTTTGTGATTCT-3' |
| *CISD1* | Forward | 5'-CCTTCACATCCAGAAAGACAACC-3' |
|  | Reverse | 5'-CTCTTCGTTATGTTTTGTGTGAGC-3' |
| *EGR1* | Forward | 5'-CTGCGACATCTGTGGAAGAAA-3' |
|  | Reverse | 5'-TGTCTGCTTTCTTGTCCTTCTG-3' |
| *HMOX1* | Forward | 5'-TCAGGCAGAGGGTGATAGAAG-3' |
|  | Reverse | 5'-TTGGTGTCATGGGTCAGC-3' |
| *CYBB* | Forward | 5'-TGGAGTTGTCATCACGCTGTG-3' |
|  | Reverse | 5'-CTGCCCACGTACAATTCGTTC-3' |
| *NOX4* | Forward | 5'-GCAGGAGAACCAGGAGATTG-3' |
|  | Reverse | 5'-CACTGAGAAGTTGAGGGCATT-3' |
| *NOX1* | Forward | 5'-CACAAGAAAAATCCTTGGGTCAA-3' |
|  | Reverse | 5'-GACAGCAGATTGCGACACACA-3' |
| *SLC11A2* | Forward | 5'-TGCTTGGTGGCCTAAAACTC-3' |
|  | Reverse | 5'-CCCCTGACAAAACCAGTCAT-3' |
| *β-ACTIN* | Forward | 5'-AGATGTGGATCAGCAAGC-3' |
|  | Reverse | 5'-TCATCTTGTTTTCTGCGC-3' |
